# Structural projections to the nucleus accumbens link to impulsive components of human risk preference

**DOI:** 10.1162/imag_a_00344

**Published:** 2024-11-05

**Authors:** Loreen Tisdall, Kelly MacNiven, Josiah Leong, Renato Frey, Jörg Rieskamp, Ralph Hertwig, Brian Knutson, Rui Mata

**Affiliations:** Center for Cognitive and Decision Sciences, University of Basel, Basel, Switzerland; Department of Psychology, Stanford University, Stanford, CA, United States; Department of Psychological Science, University of Arkansas, Fayetteville, AR, United States; Cognitive and Behavioral Decision Research, University of Zurich, Zürich, Switzerland; Center for Economic Psychology, University of Basel, Basel, Switzerland; Center for Adaptive Rationality, Max Planck Institute for Human Development, Berlin, Germany

**Keywords:** risk, reward, impulsivity, psychometric modeling, tractography, diffusion-weighted imaging

## Abstract

Functional responses in the Nucleus Accumbens (NAcc) to risk- and reward-relatedcues can predict real-life risk-taking behavior. Since NAcc activity depends onneurotransmission from connected brain regions, projections to the NAcc may alsopredict risk preference. To quantify risk preference, we employed latentvariables previously derived in a comprehensive, independent study examining thepsychometric structure of risk preference, which yielded a general riskpreference factor as well as several specific factors, including a factorcapturing impulsivity. Informed by previous work, we preregistered a set ofhypotheses concerning the association between different risk preference factorsand fractional anisotropy (or FA, which is sensitive to fiber coherence) forprojections to the NAcc from Medial PreFrontal Cortex (MPFC), Anterior Insula,Amygdala, and an inferior tract from the Ventral Tegmental Area (iVTA). Wetested our hypotheses in a community sample of 125 healthy human adults. Aspredicted, bilateral iVTA-NAcc tract FA showed a negative correlation with apsychometric factor that captured impulsivity, generalizing findings from priorresearch. Also as predicted, FA of the bilateral Amygdala-NAcc tract waspositively associated with the impulsivity factor. Contrary to predictions,however, we observed no robust associations between the general risk preferencefactor and FA for projections from bilateral MPFC, right Anterior Insula, orbilateral Amygdala to the NAcc. Notably, exploratory unilateral analysesrevealed an association between the general risk preference factor and leftMPFC-NAcc tract FA. Taken together, these findings suggest that impulse controlas a facet of risk preference maps onto specific neurobiological targets, whilemore general facets of risk preference may be supported by structural propertiesof lateral fronto-striatal projections. Although the exact associated functionalmechanisms remain to be fully clarified, conNAcctomic approaches like the onepresented here could pave the way for further research into the physiologicalfoundations of risk preference and related constructs.

## 1 Introduction

Decisions shape the start, course, and end of our lives. While some decisionscenarios are well defined and lead to trivial choices, others involve choosingbetween multiple uncertain, potentially consequential alternatives, that is, theyinvolve risk (Aven, 2012;Schonberg et al., 2011). Risk preference—thetendency to engage in potentially rewarding activities that have a probability ofharm or loss (Nigg, 2017)—and itsunderlying psychological traits are thought to be consequential for many domains oflife, including wealth, health, criminal activity, and overall well-being (Moffitt et al., 2011;Steinberg, 2013). Risk preference, thus, presents adesirable target for intervention (Conrod et al.,2013), with debates about the nature of risk and the determinants ofindividuals’ risk propensity unfolding in various disciplines, includingpsychology, economics, and biology (Bernoulli,1954;Mata et al., 2018;Mishra, 2014;Slovic, 1964).

Recent multivariate genetic analyses have started to examine potential biologicalpathways, showing that genetic differences can explain common variance in riskpreference, and, importantly, that genetic differences are predominantly expressedin brain tissue (Karlsson Linnér et al.,2021). Honing in on specific neural targets, empirical studies suggestedthat functional activation in the nucleus accumbens (NAcc) in response to reward andrisk-related cues can predict real-life risk taking (Leong et al., 2016;Sherman et al.,2018), and, in a more applied context, drug-cue-related NAcc activationhas been shown to predict relapse to stimulant use disorder (MacNiven et al., 2018). Comparative animal studies (Haber & Knutson, 2010) as well as humananatomical research (Cartmell et al., 2019)have demonstrated that the NAcc is a highly connected brain region, leading to thegeneral question whether characteristics of tracts projecting to and modulatingsignal in the NAcc—which we collectively refer to as the conNAcctome (Tisdall et al., 2022)—may be associatedwith individual differences in risk preference. The conNAcctome encompassesdopaminergic and glutamatergic tracts converging on the NAcc from the MedialPrefrontal Cortex (MPFC), Anterior Insula (AIns), Amygdala (Amy), and a tract fromthe Ventral Tegmental Area traversing below the Anterior Commissure (iVTA). Speakingto the importance of a conNAcctomic approach, dysregulation within ascendingdopaminergic projections from the midbrain to the striatum due to reduced dopamineautoreceptor availability, for example, has been demonstrated to account forindividual differences in impulsivity (Buckholtz etal., 2010).

Until recently, studying these tracts involved invasive methods (Chung & Deisseroth, 2013;Jbabdi et al., 2013;Leuze et al., 2021) unsuitable for most human research, butDiffusion-weighted Magnetic Resonance Imaging (DMRI) offers a noninvasive method toreliably reconstruct and characterize these projections in vivo (Alexander et al., 2019;Kai et al., 2022;Lazari & Lipp,2021). DMRI involves modeling and quantifying the diffusivity (i.e.,directedness) of water molecules in the brain which may support inferences about thestructure of underlying fiber bundles. Diffusion is traditionally summarized bystandard yet distinct metrics, including fractional anisotropy (FA) and radialdiffusivity (RD) (Lazari & Lipp, 2021;Suchting et al., 2021). In animal models,the reduction of lipids has been shown to causally and predictably influence DMRImetrics (e.g., lower FA and higher RD;Janve et al.,2013;Leuze et al., 2017;Ou et al., 2009;Song et al., 2002), suggesting that these metrics canpartially index myelination or lipid coherence. FA has, thus, commonly been used asan index that is sensitive to fiber coherence, with higher coherence implying moreeffective signal transmission between regions. In turn, RD (and its inverse) hasalso been linked to varying microstructural tissue properties, including axonmyelination (Beard et al., 2019;Janve et al., 2013;Song et al., 2002), fiber spread (Choe et al., 2012), and cell and axon density (Stolp et al., 2018).

The literature on DMRI approaches offers strong support for the contribution ofconNAcctome tracts to risk-related phenotypes. For example, reduced FA infronto-limbic white matter was associated with future risk taking in substance-usingadolescents (Morales et al., 2020). Moreover,right AIns-NAcc tract FA has been associated with incentivized inhibition (Leong et al., 2018) and preference for skewedgambles (Leong et al., 2016), and waspredictive of relapse to stimulant drug use (Tisdallet al., 2022). Furthermore, projections from the Amygdala have beensuggested to be associated with risk tolerance (Junget al., 2018). Finally, reduced FA of the iVTA-NAcc tract has been shownto be associated with impulsivity (MacNiven et al.,2020) and with stimulant use disorder diagnosis but not relapse (MacNiven et al., 2020;Tisdall et al., 2022).

Although these recent findings offer novel insights and, importantly, may pave theway for intervention and prevention efforts (Poldrack et al., 2018;Shivacharan etal., 2022), one remaining challenge to progress stems from the diversityof existing research when it comes to assessing risk preferences. In practice,researchers adopt different methods to capture individual differences in riskpreference: self-report measures have a long tradition in psychological research(Krosnick et al., 2005), and are used tocapture stated preferences (Frey et al.,2021;Mata et al., 2018), whilebehavioral measures with firm roots in economics reveal preferences from gamified,lottery-type tasks (Appelt et al., 2011;Beshears et al., 2009;Charness et al., 2013;Frey et al., 2021;Mata et al.,2018). In practice, different measures have repeatedly been shown to benot or only weakly correlated, with particularly behavioral measures of riskpreference and related constructs showing low convergence (Frey et al., 2017,^2021^;Mamerow et al., 2016;Mata et al., 2011,^2018^;Pedroni et al.,2017). As such, we would expect the results of studies probing the linkbetween brain tract structure and risk preference to vary as a function of how riskpreference was operationalized. This makes the identification of reliable neuraltargets for intervention a challenging endeavor.

In this study, we aimed to tackle the issue of measurement plurality and theresulting divergence of brain-outcome associations. Specifically, we aimed to avoidbiased estimates stemming from the use of single measures of risk preference byemploying risk preference factors as our outcome variables. These factors werederived independently through psychometric modeling based on 39 risk-taking measurescollected in a sample of over 1500 young adults (Frey et al., 2017). Combining psychometric risk preference factors witha conNAcctomic approach, our goal was to examine whether coherence-sensitive DMRImetrics of specific brain tracts relate to individual differences in riskpreference.

Recent work (Marek et al., 2022) has suggestedthat when searching brainwide for brain-behavior associations, a sample size ofhundreds or thousands of participants is required to establish robust associations,and relevant genetic associations for risk-related outcomes (Aydogan et al., 2021) have been reported for very largedatasets. Sample size is of critical importance for the internal validity of astudy, primarily through its impact on the reliability and precision of the results,as well as the ability to detect true effects. In this study, we sought to increaseinternal validity by (a) carefully selecting brain and behavioral indices withmoderate to high test-retest reliability (cf.section2), (b) focusing on a small, a priori defined set of brain regionspreviously implicated in the outcomes under investigation, and (c) exhaustivelycontrolling for confounds. In addition, we preregistered a set of hypothesesconcerning the associations between brain tract structure and risk preferencefactors (Table 1).

**Table 1.** Research hypotheses for associations between risk preference and conNAcctomestructure (FA and 1/RD).

H	Factor	Tract (hemisphere)	Predicted association	Selected source for prediction
1	R	MPFC-NAcc (lr)	−	Morales et al. (2020)
2	R	AIns-NAcc (r)	−	Leong et al. (2016 , 2018 )
3	R	Amy-NAcc (lr)	±	Cohen et al. (2009) ; Jung et al. (2018)
4	F4	Amy-NAcc (lr)	+	van den Bos et al. (2014)
5	F4	iVTA-NAcc (lr)	−	MacNiven et al. (2020)

*Note.*H = Hypothesis; r = right; lr= bilateral.

We based our hypotheses on not only previous empirical findings but also mechanisticexplanations, including the idea that decreased FA between the right AIns and NAccmight result in the decreased dampening of NAcc-related reward signal as a functionof an affective inhibitory signal originating in AIns (Leong et al., 2016,^2018^). For example, we predicted a negative association between ageneral risk preference factor (R) and bilateral MPFC-NAcc FA (H1), and with rightAIns-NAcc FA (H2). We also predicted an association between R and bilateral Amy-NAccFA (H3), but tested a bidirectional hypothesis due to the heterogeneity of previousfindings. For F4, a factor capturing impulsivity, we predicted a positiveassociation with bilateral Amy-NAcc FA (H4) but a negative association withbilateral iVTA-NAcc FA (H5).

## 2 Method

The study was reviewed and approved by the German Society for Psychology, and theEthics Committee of the Max Planck Institute for Human Development. All methods werecarried out in accordance with the relevant guidelines and regulations. Prior toparticipation in the study, all individuals gave written informed consent. Theanalyses were preregistered on AsPredicted (https://aspredicted.org/bx49i.pdf).

### 2.1 Participants

The participants in this neuroimaging study were recruited from an existing poolof individuals who had taken part in the Basel-Berlin Risk Study (BBRS). Theoverarching aim of the BBRS is to examine the psychometric structure andbiological underpinnings of risk preference in a large sample (N = 1507)of young human adults (Dutilh et al.,2017;Frey et al., 2017;Pedroni et al., 2017;Tisdall et al., 2022). Participation in the BBRSinvolves completion of a laboratory session, during which individuals areassessed on a large battery of behavioral and self-reported risk-takingmeasures, as well as on other individual differences measures, includingcognitive capacity, personality, affect, and genetics. Further details andsummaries of all BBRS sub-samples and measures are reported on the BBRS OSFrepository (https://osf.io/rce7g).The BBRS was run in Basel (Switzerland) and in Berlin (Germany); for this studywe only recruited individuals from the Berlin site due to the location of thescanning facilities available. Exclusion criteria for participation were anycontraindications with regards to magnetic resonance imaging (MRI) safety (e.g.,safety-limiting non-removable implants), a history of neurological orpsychiatric conditions, reported use of psychoactive medication or substances,and concurrent psychiatric treatment.

To compensate for participant exclusions (e.g., due to excessive head motion inthe scanner, image artifacts), we recruited a total of 133 participants toachieve a minimum effective sample size of N∼100 participants (Yarkoni, 2009). Twoparticipants aborted the scanning session before any MRI data were collected;these two participants were removed from all subsequent analyses. A further fiveparticipants were excluded because the critical diffusion-weighted imagingsequence necessary for this project was not collected due to time constraints,and one additional participant was excluded due to incidental anatomicalfindings. After exclusions, all analyses included a sample of 125 young adulthuman participants comprising 67 (53.6%) females, with a sample mean age of25.19 years (SD = 2.57, range = 20.4–30.1 years).

### 2.2 Study protocol

We contacted individuals who had previously completed the BBRS laboratory sessionby phone and invited them to a follow-up neuroimaging study. Interestedindividuals were initially screened for any MRI-safety contraindications priorto being enrolled in the study, and again on the day of the MRI session. Allparticipants provided written informed consent. Participants were then scannedusing MRI to acquire anatomical and diffusion-weighted images. We also collectedfunctional scans, but these do not contribute to the current analyses and arenot described further (cf.Tisdall et al.,2020). Due to the fact that participants in the MRI session werescanned at different intervals after completing the laboratory component (medianinterval = 6.28 months, mean interval = 6.63 months, SD =3.99 months, range = 1–453 days), we performed supplementaryrobustness checks to control for interval.

After scanning, participants completed a demographic questionnaire as well asadditional measures that we do not focus on in the current analyses.Participants received a base payment of 25 Euro for their participation. Inaddition, participants could increase their earnings based on their decisionmaking in the two functional MRI tasks. All participants were informed about theincentive structure and received cash earnings at the end of the session (meantotal earnings = 41.50 Euro, SD = 14.50 Euro).

### 2.3 Risk preference factors

The quantification of risk preference based on single risk-taking measures, inparticular behavioral measures, runs into problems of validity, reliability, andconvergence (Enkavi et al., 2019;Frey et al., 2017;Pedroni et al., 2017). To address these issues, weused risk preference factors as outcome variables that had previously andindependently been derived from the psychometric modeling of 39 risk-takingmeasures collected in the full BBRS sample (Freyet al., 2017). Notably, the BBRS included three broad categories ofmeasures, namely propensity, frequency, and behavioral measures; seeSupplementary MethodsandTable S1fordetails concerning specific risk preference measures and the adopted bifactormodel. The implemented bifactor model extracted a general risk preferencefactor, R, and seven orthogonal specific factors which captured additionaldomain- or situation-specific variance. These domain-specific factors werethought to capture attitudes and behaviors associated with health risk taking(F1), financial risk taking (F2), recreational risk taking (F3), impulsivity(F4), traffic risk taking (F5), occupational risk taking (F6), and choices in(monetary) lotteries (F7).

**Fig. 1.**
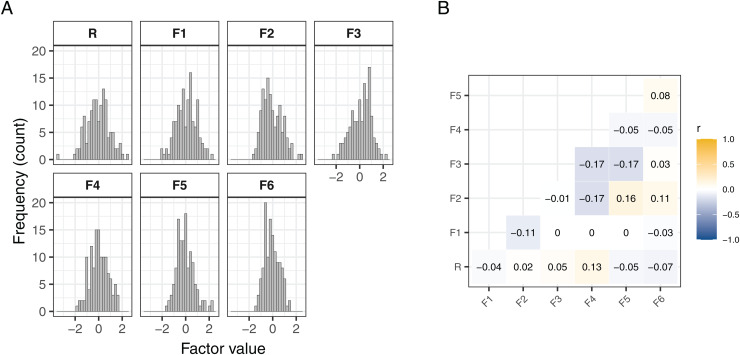
Risk preference factors. (A) Distribution of the general anddomain-specific risk preference factors. (B) Correlation analysesbetween the psychometric factors for the MRI sub-sample of the BBRS (N= 125) confirms orthogonality between the factors. R =General, F1 = Health, F2 = Financial, F3 =Recreational, F4 = Impulsivity, F5 = Traffic, F6 =Occupational.

Test–retest reliability for the psychometric factors (assessed in asub-sample of 106 BBRS laboratory participants) was shown to be higher than forany of the behavioral measures; for example, R had a 6-month retest reliabilityof 0.85, whereas many of the behavioral measures tested yielded retestreliabilities below 0.5. In this study, we focused on factors with test-retestreliabilities above 0.5, that is, R and all domain-specific factors except F7.Our main hypotheses concerned R (test-retest correlationr=0.85) and the impulsivity-capturing factor F4 (test-retestcorrelationr=0.7), but we also conducted exploratory analyses on the remainingdomain-specific factors. Factor values for the current MRI sample wereapproximately normally distributed (Fig. 1,panel A;SupplementaryMaterials) and mainly orthogonal (Fig.1, panel B).

### 2.4 Scan acquisition

Neuroimaging data were collected at the MRI Laboratory at the Max PlanckInstitute for Human Development (Berlin, Germany) on a 3T Siemens MRI systemwith a 12-channel head coil. For the DMRI, we used a single-shot echo-planarimaging sequence with the following parameters: b = 1000s/mm^2^, 61 diffusion directions, TR = 10 s, TE = 94 ms,fat saturated flip angle = 110°, FOV = 208 mm x 208 mm, 69axial slices, and voxel dimensions = 2.0 mm isotropic. At the beginningof the DMRI scan, we acquired eight non-diffusion weighted images (b = 0s/mm^2^). A structural T1-weighted scan was acquired for everyparticipant at the start of the MRI session via a magnetization-prepared rapidgradient echo sequence (repetition time = 2500 ms, echo time =4.77 ms, inversion time = 1100 ms, flip angle = 7°, FOV= 256 mm × 256 mm, 192 slices, voxel dimensions = 1.0 mmisotropic).

### 2.5 ConNAcctome volumes of interest (VOI)

**Fig. 2.**
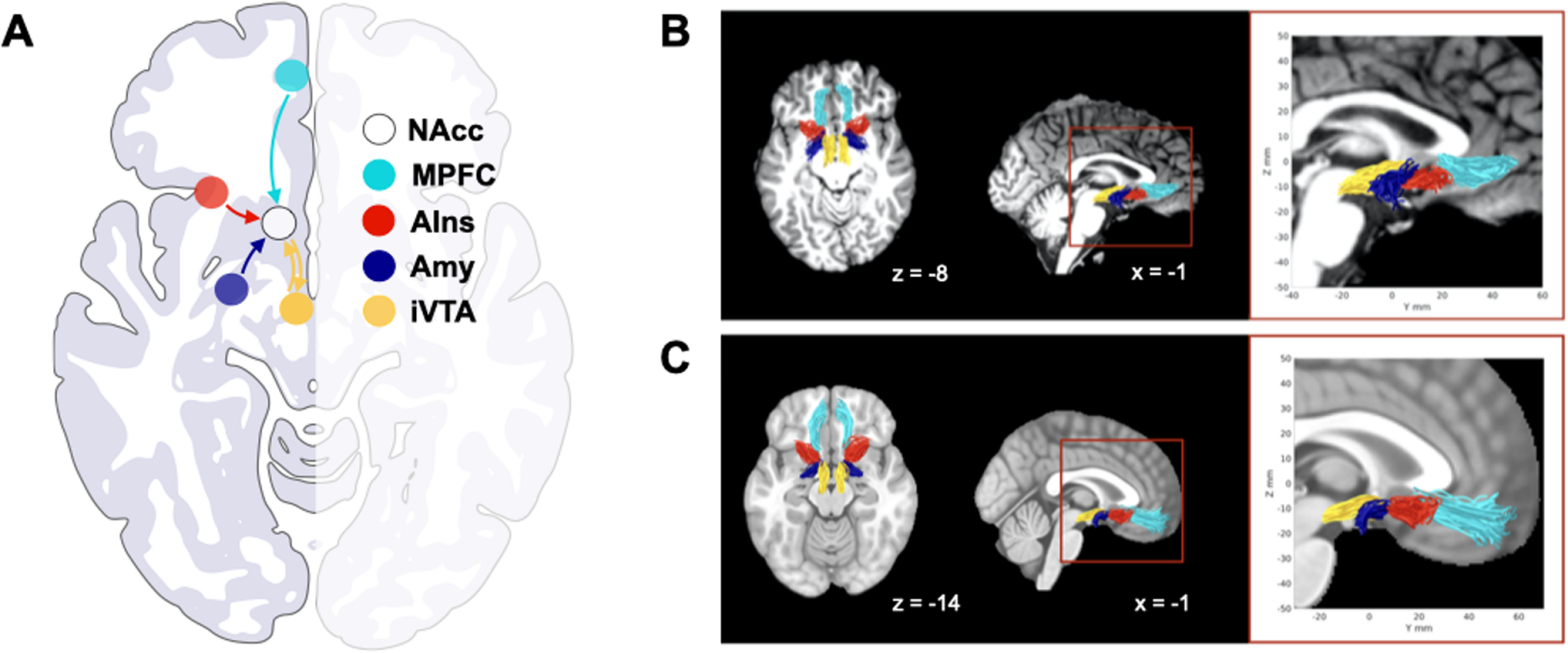
ConNAcctome tracts. (A) Schematic with previously demonstrateddirectionality of projections. (B) Tracts in a representativesubject’s AC-PC aligned native brain space. (C) Group tracttemplates in MNI space.

We focused on four tracts projecting to the NAcc (Fig. 2), namely from the MPFC, AIns, Amy, and Ventral Tegmental Area(VTA) (Haber & Knutson, 2010). Forthe seed-based tractography in individuals’ native space, we defined theNAcc, AIns, and Amy VOIs based on automated tissue segmentation and parcellationusing FreeSurfer (Leong et al., 2016,^2018^;MacNiven et al., 2020;Tisdall etal., 2022), and the MPFC and VTA VOIs were defined based onpreviously reported methods (Leong et al.,2016;MacNiven et al., 2020;Samanez-Larkin et al., 2012;Tisdall et al., 2022). We also created anative space white matter mask for every participant (using FreeSurfer); thiswas used to restrict tractography to white matter voxels. Previous worksuggested anatomically-specific associations between impulsivity and a VTA tracttraversing below (versus above) the Anterior Commissure (AC) (MacNiven et al., 2020;Tisdall et al., 2022). To examine this specificity,we performed tractography for the VTA-NAcc tract using an exclusionary mask atthe AC and thereby isolated separate fiber bundles projecting from the VTA tothe NAcc, which run below and above the AC. This allowed us to separatelycharacterize the superior and inferior VTA (iVTA) tracts, and examine theirassociation with impulsivity. In our schematic of the conNAcctome (Fig. 2, panel A), we include directionalpathways between brain regions. While the current approach using diffusion MRIcannot resolve directionality, comparative work (e.g., using tracer studies) hasenabled identification of afferent and efferent connections within the rewardcircuitry (Haber & Knutson, 2010).Thus, our schematic incorporates these insights.

### 2.6 DMRI data preprocessing and tractography

The raw DMRI data were preprocessed using the open-source software packagemrDiffusion (www.github.com/vistalab/vistasoft), which we ran in MATLAB R2016b. Asdescribed in (MacNiven et al., 2020;Tisdall et al., 2022), preprocessingincluded motion correction, registration of each diffusion-weighted image to themean of the non-diffusion weighted (b = 0) images, co-registration of themean of the non-diffusion weighted (b = 0) images to the T1-weightedvolumes, and resampling of the raw diffusion-weighted images to 2 mm isotropicvoxels (Ashburner & Friston, 2005).Using least-squares error minimization, we fit tensors to the diffusionmeasurements in each voxel. From each voxel’s tensor, we then createdvoxel-wise maps of Fractional Anisotropy (FA), Mean Diffusivity (MD), RadialDiffusivity (RD), and Axial Diffusivity (AD). To precisely identify andcharacterize the conNAcctome tracts, we combined probabilistic tractography inindividuals’ native space with constrained spherical deconvolution usingMRtrix (v3.0) (Tournier et al., 2007).Application of a constrained spherical deconvolution model of water diffusionallowed us to model crossing fibers. In a first step, we fit a model with amaximum harmonic envelope of 8 to estimate the Fiber Orientation Distribution(FOD) within each voxel. In a second step, we tracked fiber pathways between theseed VOIs and the NAcc, using the FOD as a probability density function (in eachvoxel). The tractography was performed using the following parameters: desirednumber of fibers = 1000; maximum number of attempted fibers = 107;algorithm, iFOD2; and FOD cutoff = 0.05. Using participants’ whitematter masks, tractography was restricted to white matter voxels only. Thereconstructed fiber groups were subsequently cleaned with Automated FiberQuantification (Yeatman, Dougherty, Myall, etal., 2012). For this, fiber groups were resampled to 100 equidistantnodes between the seed and NAcc VOIs. We then calculated the core of the fibergroup as the mean coordinates at each of the 100 nodes. Fibers with coordinatesthat fell more than three standard deviations from the core coordinates of thefiber group (via computation of the Mahalanobis distance) were excluded from thefiber group, as were fibers with a length more than two standard deviations fromthe mean length of the fiber group. Cleaning was performed in an iterativefashion with a maximum of five iterations. To compare fiber density maps acrosssubjects in tract segmentation analyses, each subject’s anatomical imagewas spatially normalized to a group template (TT_N27) using a non-linearregistration method with ANTS software (Avants etal., 2014).

### 2.7 DMRI metrics

Our statistical analyses targeted FA and RD; for ease of interpretation, wecalculated the inverse of RD (1/RD) to align with the notion of a higher metricbeing indicative of more fiber coherence (cf.Supplementary Materialsfor further details). Initial plotting of node-wise FA and 1/RD for all 125participants identified one participant with outlier tract metrics for theMPFC-NAcc tract (cf.Supplementary Materialsfor further details on outlier detection);we excluded this particular participant from analyses involving the MPFC-NAcctract. Critically, extant work conducted by the current authors as well asexternal researchers (Kruper et al.,2021;Rokem et al., 2015;Tisdall et al., 2022) suggests thatdiffusion metrics show moderate to (very) high reliability, thus reducingmeasurement error and contributing to internal validity.

### 2.8 Analysis protocol for individual differences analyses

Our analyses targeted out-of session associations between psychometric riskpreference factors and structural projections. Following previously establishedanalytical routines (Tisdall et al.,2022), we adopted the following order of analytical procedures to testour hypotheses.

First, for every hypothesis, we followed previously described methods (Nichols & Holmes, 2002) and adopteda linear regression-based permutation test approach to establish the requirednumber of consecutive nodes along a given tract for an association to besignificant at p<0.05 (corrected for multiple testing along a tract of 100 nodes). This approachis comparable to computing a cluster extent for functional MRI analyses.

Second, we used linear regression analysis to identify all nodes along a giventract which showed an association at p<0.05 (uncorrected) with the specific risk preference factor ofinterest, and compared the number of observed consecutively significant nodeswith the number of required consecutively significant nodes.

Third, for the purpose of model comparison, for every node cluster that met orexceeded the required number of consecutively significant nodes, we calculated atract-specific summary FA (or 1/RD) metric on the basis of a simple average overall observed consecutively significant nodes, and used this summary tract metricin multiple linear regression analyses. We note that analyses using summarytract metrics are not independent because the selection of nodes is informed bythe results from the node-wise analyses (Vul& Pashler, 2012), hence they do not provide unbiasedestimates. However, these analyses do not challenge the main (node-wise)results, and facilitate both controlling for confounds and comparison withprevious work. All regression analyses were based on standardized andresidualized (with regards to age and gender) risk preference factor values, aswell as standardized summary tract metrics to facilitate effect comparisonacross tracts.

Fourth, to estimate and visualize the association between tract metrics and riskpreference factors while exhaustively controlling for the influence ofdemographic (age, gender) and methodological (i.e., number of streamlines)variables, we adopted a multiverse approach (Steegen et al., 2016) to data analysis by performing specificationcurve analysis (Frey et al., 2021;Simonsohn et al., 2020) using the summarytract metrics (cf.Supplementary Materialsfor details). We followed up on the mainanalyses with exploratory analyses aimed at examining potential lateralityeffects, and to pinpoint additional associations which may be targeted moredirectly in future studies.

## 3 Results

### 3.1 ConNAcctome tracts

We successfully tracked and characterized all tracts bilaterally in all subjects.Figure 2, panel B shows the conNAcctometracts in an individual’s AC-PC aligned native brain space, illustratingthe anterior-to-posterior location of the tract endpoints in the NAccoriginating in the MPFC, AIns, Amy, and iVTA, respectively. To visualize andqualitatively compare tract locations across subjects, for each tract wegenerated group templates in standard MNI space (Fig. 2, panel C). The MNI group tracts suggest a high degree ofsimilarity across subjects concerning the location of conNAcctome tracts in bothhemispheres. We plotted sample and individual tract profiles for FA and 1/RDseparately for the two hemispheres of each tract (Fig. S1). As describedpreviously (Yeatman, Dougherty, Ben-Shachar,& Wandell, 2012), tract metric profiles were heterogeneousacross tracts, but comparatively more homogeneous across hemispheres.

### 3.2 Risk preference as a function of tract metrics

#### 3.2.1 Node-wise associations

**Fig. 3.**
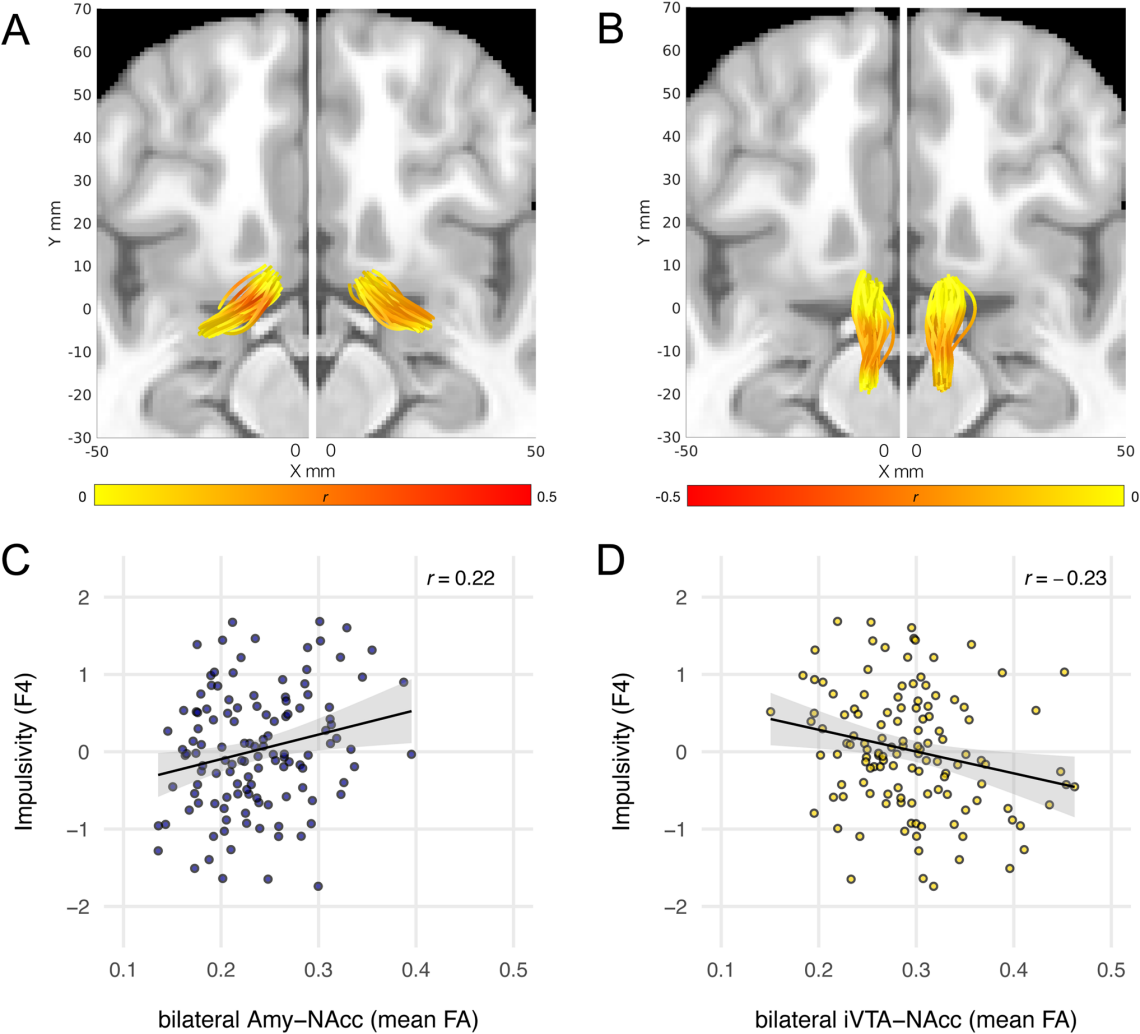
Association between impulsivity (F4) and FA. (A, B) Group tracts inMNI space with superimposed node-wise associations betweenimpulsivity and FA in left and right Amy-NAcc tract (A), and FA inleft and right iVTA-NAcc tract (B). Each tube represents the coretrajectory of one participant’s tract (in MNI space). Colorsindicate node-wise correlation coefficients. (C) Bivariateassociation between impulsivity and bilateral Amy-NAcc mean FA(calculated from significant nodes shown in panel A). (D) Bivariateassociation between impulsivity and bilateral iVTA-NAcc mean FA(calculated from significant nodes shown in panel B).

For our main individual differences analyses, we followed a permutation-basedapproach to evaluate node-wise associations between tract metrics and riskpreference factors while controlling for multiple comparisons (see Methodsfor details). As predicted, FA of the Amy-NAcc tract (bilateral) waspositively associated with F4 (Table2;Fig. 3, panel A); thisresult was specific to FA and did not extend to 1/RD (Table S2;Fig. S4). Also aspredicted, we observed a negative association between theimpulsivity-capturing factor F4 and FA in an extended cluster of nodes alongthe bilateral iVTA-NAcc tract (Table2;Fig. 3, panel B). Thisassociation was also observed for 1/RD (Table S2;Fig. S4).

**Table 2.** Node-wise regression results for FA, organized by hypothesis.

H	Factor	Tract (hemisphere)	# Observed/# required	Nodes	Predicted direction	Observed direction
1	R	MPFC-NAcc (lr)	16/27	27–42	−	+
2	R	AIns-NAcc (r)	10/25	33–42	−	+
3	R	Amy-NAcc (lr)	0/32	n/a	+	n/a
**4**	**F4**	**Amy-NAcc (lr)**	**32/31**	**49–80**	+	+
**5**	**F4**	**iVTA-NAcc (lr)**	**32/26**	**23–54**	−	−

*Note.*H = Hypothesis; # Observed/#Required = Number of observed versus number of requiredconsecutively significant(p=0.05)nodes; lr = bilateral; r = right;−= negative;+= positive;±= bidirectional; bold font =#Observed>#Required.

Although we identified node clusters in bilateral MPFC-NAcc and rightAIns-NAcc associated with the general risk preference factor R (Fig. S4), theextent of these clusters did not meet the required threshold, and theobserved associations were contrary to the predicted directions. Contrary toour predictions, we found no association between the general risk preferencefactor R and Amy-NAcc FA or 1/RD (Table2;TableS2).

#### 3.2.2 Model comparison

To examine the robustness of the observed brain-behavior associations, weperformed a set of model comparisons. For this purpose, we generated summarytract metrics for the bilateral Amy-NAcc and iVTA-NAcc tracts, by averagingFA metrics across the consecutive nodes found to be significantly associatedwith the impulsivity-capturing factor F4 (cf. ‘Nodes’ columninTable 2), and used these in modelcomparisons. The scatterplots inFigure3show the distribution of the resultant summary tract metricsand provide an intuitive visualization of the association between F4 andbilateral Amy-NAcc (panel C) and bilateral iVTA-NAcc (panel D) mean FA. Tonote, due to the dependence of the summary metrics on the node-wiseanalyses, the Pearson correlation coefficients reported in panels C and D donot provide unbiased estimates.

To understand whether the two tracts make independent contributions to theimpulsivity-capturing factor F4, we performed a set of linear regressionmodels. The results suggest that FA of the Amy-NAcc tract and iVTA-NAcctract make independent contributions to explaining variance in F4, and thatthese contributions remain when we control for demographic andmethodological covariates (Table 3).Furthermore, specification curve analyses (Fig. S2) supported(a) the robustness of the observed associations between F4 and FA of bothAmy-NAcc and iVTA-NAcc projections against the inclusion of exhaustive setsof covariates, and (b) the specificity of the observed results for F4, butnone of the other risk preference factors. Supplementary analyses indicatedno effect of interval between the laboratory and MRI session on the mainresults (Fig.S3).

**Table 3.** Linear regression models for impulsivity (F4).

Variable	Demographic	Amy-NAcc	iVTA-NAcc	Combined
Intercept	0.04 (0.09)	0.02 (0.07)	0.02 (0.07)	0.09 (0.09)
Age	-0.12 (0.07)	—	—	**-0.13 (0.07)**
				**[-2.01, 0.047]**
Gender (male)	-0.04 (0.14)	—	—	-0.14 (0.14)
Amy-NAcc (nsl)	—	0.02 (0.07)	—	0.01 (0.07)
iVTA-NAcc (nsl)	—	—	0.03 (0.07)	0.07 (0.07)
Amy-NAcc (FA)	—	**0.18 (0.07)**	—	**0.17 (0.07)**
		**[2.56, 0.012]**		**[2.47, 0.015]**
iVTA-NAcc (FA)	—	—	**-0.17 (0.07)**	**-0.15 (0.07)**
			**[-2.54, 0.012]**	**[-2.11, 0.037]**
AIC	295.07	291.72	291.56	290.12
Adjusted ${\text{R}}^{2}$	0.01	0.04	0.04	0.08
RSS ( p )	72.55 (0.008)	70.63 (0.035)	70.55 (0.037)	64.94 (—)

*Note.*Estimates represent standardizedregression coefficients. We report the standard error of theestimate in round brackets. For significant variables(p<0.05),we report the exact t-value and p-value in square brackets.Gender was coded with female as the reference category, thusestimates are for males relative to females. nsl = numberof streamlines. RSS = residual sum of squares andassociated probability(p)for comparison of nested model to full (combined) model. Boldfont = p<0.05.

#### 3.2.3 Exploratory analyses

Beyond testing for the hypothesized associations, we also performedexploratory analyses to better understand potential laterality effects(specifically for tracts for which we aggregated tract metrics acrosshemispheres to test bilateral hypotheses), and to gauge further associationsthat may present promising starting points for future research (Fig. S4). Withregard to laterality, we observed comparably strong effects in left andright hemispheres for the associations between impulsivity and both theiVTA-NAcc and Amy-NAcc tracts. Replicating previous work (MacNiven et al., 2020), supplemental analysesconfirmed the anatomical specificity of the link between impulsivity andiVTA-NAcc tract, as no association was observed for the superior tractprojecting from the dopaminergic midbrain to the NAcc. Supplementaryanalyses suggested a strongly lateralized effect of MPFC-NAcc FA on thegeneral risk preference factor R, which we examined further in exploratorysupplemental analyses (cf.Supplementary Results). In addition, we observed alateralized effect of MPFC-NAcc FA for F1, a factor capturing health-relatedbehaviors (TableS3).

## 4 Discussion

The central question of this research was whether structural characteristics ofwhite-matter fiber tracts converging on the NAcc are associated with individualdifferences in human risk preference. Previous studies have suggested thatfunctional responses in the NAcc to risk- and reward-related cues can predictreal-life risk-taking behavior (Leong et al.,2016;Sherman et al., 2018),account for impulsivity (Buckholtz et al.,2010) and predict drug relapse (MacNivenet al., 2018). Given that NAcc functional activity is driven byneurotransmission from connected brain regions, we hypothesized that DMRI metrics ofconNAcctome tracts—including projections from the MPFC, AIns, Amy, andiVTA—may also account for individual differences in risk preference. Tocounter biased estimates as a result of adopting single, often unreliable riskpreference measures (Enkavi et al., 2019;Frey et al., 2017,^2021^;Tisdall et al.,2020), here we defined our outcome variables via risk preference factorsderived through psychometric modeling in independent research (Frey et al., 2017).

Our results reinforce the notion of the modulatory role played by differentprojections to the NAcc for specific components of risk preference. Extendingprevious work (MacNiven et al., 2020) andsupporting our prediction, we observed a robust negative association betweenF4—a factor capturing impulsivity—and FA (as well as 1/RD) of a tractprojecting from the dopaminergic midbrain (iVTA) to the NAcc. This link remainedwhen we included demographic and methodological covariates in our analyses, and wasspecific to a projection traversing below (as opposed to above) the AC (MacNiven et al., 2020). Critically, theobserved negative association between impulsivity and iVTA-NAcc FA (and 1/RD)demonstrates generalizability of previous findings (MacNiven et al., 2020;Tisdall et al.,2022) to an independent, non-US sample, thus pointing towards a robustfinding in need of a physiological explanation. Regarding their functional role, FAand RD have been shown to capture microstructural properties (e.g., axonmyelination) (Choe et al., 2012;Janve et al., 2013;Leuze et al., 2017,^2021^;Ou et al., 2009;Song et al., 2002) of brain tracts, which mightaffect signal transfer; if higher FA indexes more (coherent) myelination, this couldfacilitate more efficient or reliable transfer of chemical (e.g., neurotransmitter)and electrical signals between connected brain regions. Suggestive of an endogenousdopamine-related mechanism, prior research has shown that sustained dopamine releasein the striatum plays a role in mediating the negative association between dopaminereceptor availability in the midbrain and trait impulsivity (Buckholtz et al., 2010); whether iVTA-NAcc tractcoherence contributes to this pathway, or supports a separate pathway, requiresfurther research.

Also in line with predictions, and independently of the effect for iVTA-NAcc,Amy-NAcc FA was positively associated with F4 (vanden Bos et al., 2014). The ‘leakiness’ of signaltransmission as a function of low coherence could account for this finding. Forexample, previous work reported a positive association between impulsivity (indexedvia temporal discounting) and bilateral connection strength between the amygdala andthe striatum (van den Bos et al., 2014),which the authors suggested may have been driven by increased input from theamygdala—computing an incentive value of immediately availableoptions—enhancing the value signal in the striatum.

While these results present a generalization of previous findings (MacNiven et al., 2020) to an independent sample withdifferent demographics, our impulsivity measure was similar to that used in previouswork. Specifically,MacNiven et al. (2020)reported iVTA-NAcc FA associations with impulsivity indexed by the BarrattImpulsiveness Scale (BIS) as well as a behavioral measure of impulsivity. Theimpulsivity factor F4 derived independently byFreyet al. (2017)also exclusively captures variation in BIS. While thebifactor model removed common variance across the battery of risk measures adoptedin the BBRS laboratory session from the impulsivity factor, the remaining highpositive correlations between F4 and BIS (Fig. S5) suggest that F4 can index BIS. The previouslyreported association between iVTA-NAcc FA and delay discounting (MacNiven et al., 2020) presents a first hint that thisassociation with impulsivity may extend to alternative impulsivity measures, butfurther work is required to more systematically examine the convergence ofbrain-behavior associations across different measures, both within as well as acrossmeasurement modalities and domains.

In contrast to our hypotheses concerning the impulsivity-capturing factor F4, wefound no support for our predictions concerning associations between the generalrisk preference factor and bilateral projections from the MPFC, Amy, and right AIns.This discrepancy may be driven by the extent to which (a) different psychometricfactors capture specific (cognitive, affective) versus more general, perhaps evenstrategic processes underlying individual differences (and the reporting thereof),and (b) these processes are subserved by concrete neural substrates like specificbrain tracts. For example, to the extent that F4 captures a very specific dimensionof risk preference, namely impulse control, the latter has been found to mapdirectly onto (neural markers of) motivation and reward-related mechanisms (Buckholtz et al., 2010;Dalley & Robbins, 2017;Hampton et al., 2017;MacNiven et al., 2020).

Moreover, our exploratory analyses indicated a link between right MPFC-NAcc FA and adomain-specific factor capturing health-related attitudes and behaviors, thusspeaking to the utility of examining domain-specific aspects of risk preference, andassociations in specific (e.g., clinical) cohorts (Hampton et al., 2019;MacNiven et al.,2020;Morales et al., 2020;Tisdall et al., 2022). Notably, exploratoryunilateral analyses also suggested an association between left MPFC-NAcc FA andgeneral risk preference, but given the observed positive effect as opposed to thepredicted negative effect, further research is required to replicate the currentfinding. If replicated, however, this finding could have important implications forextant and future work. For example, fibers between the MPFC and Amygdala have beenlinked to risk preference (Jung et al.,2018), yet it is possible that these reported fibers actually traversethrough the NAcc. In light of our results linking Amy-NAcc projections toimpulsivity (rather than general or other domain-specific aspects of riskpreference), and exploratory results linking MPFC-NAcc projections to generalaspects of risk preference, our results may add anatomical specificity to thesepreviously reported findings.

Our study has some limitations in need of discussion. First, our approach to utilizeindependently derived risk preference factors aimed to avoid the pitfalls of usingsingle measures with regards to reliability, validity, and convergence (Enkavi et al., 2019;Frey et al., 2017,^2021^;Mata et al., 2018;Pedroni et al., 2017). However, this approachraises the question what exactly the resultant factors represent, in particular thegeneral risk preference factor*R*, and how the variance betweenindividuals in this general factor maps onto discrete mechanisms and their neuralbasis. What is captured by*R*might be the result of many processes,including how individuals integrate aspects such as probability, gains, and losses,as well as modality-specific processes such as how questions are understood andresponses generated (note that*R*captures exclusively commonvariance across self-report measures). Although the cognitive processes underlyingthe rendering of self-reported risk preference are starting to become betterunderstood (Steiner et al., 2021), how theseare represented in the brain remains to be examined. Moving forward, conceptualclarification of the ‘essence’ of risk preference (Bringmann et al., 2022) will lie at the heart of progressin our understanding of its neurobiological basis.

Second, the desiderata for the outcome variable equally apply to the predictor(s),the brain markers. A body of work suggests that DMRI metrics are reliable (Kruper et al., 2021;Rokem et al., 2015;Tisdall et al., 2022), relate to microstructural properties (Lazari & Lipp, 2021), and are related toimportant (clinical) phenotypes (Forkel et al.,2022;Joutsa et al., 2022).However, it is currently still unclear exactly which microstructural properties(e.g., myelination, iron deposition) are captured by metrics such as diffusioncoefficients (Lazari & Lipp, 2021;Weiskopf et al., 2021) and how differentmetrics relate, that is, their convergence. The former will be important to forgeahead with intervention programs targeting specific microstructural properties(Fields, 2015), while the latter will becrucial to efficiently and meaningfully compare results across studies usingdifferent tract metrics. For example, our supplementary exploratory analysessuggested no link between impulsivity and fronto-striatal tract structure shown inprevious work (Hampton et al., 2017);however, here we used diffusion metrics while previous work computed the number ofstreamlines to index connectivity strength. To make progress, we need to map thespace of common structural metrics and their relationships. To disentangle the roleof the molecular, cellular, circuit, and system levels, it will be important toconcurrently employ a range of analytical approaches. Fortuitously, advancedneuroscientific methods including tissue clearing (Chung & Deisseroth, 2013) and optogenetic research (Cao et al., 2011) are well poised to providemuch-needed answers to questions pertaining to the mapping of metrics tomicrostructural properties to (behavioral) phenotypes.

Third, in this study, we used data from a cross-sectional sample of healthy youngadults, yet the ultimate test will involve examination of the predictive validity ofconNAcctome tracts. For this, it will become crucial to harvest existing(large-scale) data sets with longitudinal designs that include repeated scanningsessions, such as the ABCD Study (Casey et al.,2018) or Human Connectome Project (VanEssen et al., 2013). This would also allow researchers to test varioustheories relating to individual (age) differences in risk preference (Frey et al., 2021;Seaman et al., 2022), potentially even teasing apart thecausal mechanisms, such as the effects of socialization and/or selection (Beck & Jackson, 2022). This approachshould also be extended to test the predictive validity of biomarkers in differentsamples, including impulsivity-related outcomes in clinical populations (Karlsson Linnér et al., 2021;Kotov et al., 2018).

## 5 Conclusion

Illuminating the neurobiological basis of individual differences in maladaptivecognition and behavior has the potential for intervention and prevention (Shivacharan et al., 2022), but researchersfirst need to find reliable indicators for brain markers, outcome measures, andtheir association. Here, we combined a principled selection of brain tracts with apsychometric assessment of risk preference to provide a first step in this directionfor a phenotype that shapes decision making and, consequently, impacts health,wealth, safety, and overall well-being (Moffitt etal., 2011;Steinberg, 2013).Studying the role of the brain, and in particular mapping the generality andspecificity of brain markers for risk preference and related constructs, may offernew insights for intervention, and, potentially, prevention.

## Supplementary Material

Supplementary Material

## Data Availability

The main analyses reported in this manuscript were preregistered via AsPredicted(https://aspredicted.org/bx49i.pdf). Data and code supporting the mainanalyses reported in this paper are available on the Open Science Framework (https://osf.io/t27cq/). A preprintversion of this manuscript was uploaded to the Open Science Framework (https://osf.io/ab4vr/).

## References

[b1] Alexander , D. C. , Dyrby , T. B. , Nilsson , M. , & Zhang , H. ( 2019 ). Imaging brain microstructure with diffusion MRI: Practicality and applications . NMR in Biomedicine , 32 ( 4 ), e3841 . 10.1002/nbm.3841 29193413

[b2] Appelt , K. , Milch , K. , Handgraaf , M. , & Weber , E. ( 2011 ). The decision making individual differences inventory and guidelines for the study of individual differences in judgment and decision-making research . Judgment and Decision Making , 6 ( 3 ), 252 – 262 . 10.1017/S1930297500001455

[b3] Ashburner , J. , & Friston , K. J. ( 2005 ). Unified segmentation . NeuroImage , 26 ( 3 ), 839 – 851 . 10.1016/j.neuroimage.2005.02.018 15955494

[b4] Avants , B. B. , Tustison , N. J. , Stauffer , M. , Song , G. , Wu , B. , & Gee , J. C. ( 2014 ). The Insight ToolKit image registration framework . Frontiers in Neuroinformatics , 8 , 1 – 13 . 10.3389/fninf.2014.00044 24817849 PMC4009425

[b5] Aven , T. ( 2012 ). The risk concept — Historical and recent development trends . Reliability Engineering and System Safety , 99 ( 0951 ), 33 – 44 . 10.1016/j.ress.2011.11.006

[b6] Aydogan , G. , Daviet , R. , Karlsson Linnér , R. , Hare , T. A. , Kable , J. W. , Kranzler , H. R. , Wetherill , R. R. , Ruff , C. C. , Koellinger , P. D. , BIG BEAR Consortium , & Nave , G. ( 2021 ). Genetic underpinnings of risky behaviour relate to altered neuroanatomy . Nature Human Behaviour , 5 ( 6 ), 787 – 794 . 10.1038/s41562-020-01027-y PMC10566430 33510390

[b7] Beard , C. L. , Schmitz , J. M. , Soder , H. E. , Suchting , R. , Yoon , J. H. , Hasan , K. M. , Narayana , P. A. , Moeller , F. G. , & Lane , S. D. ( 2019 ). Regional differences in white matter integrity in stimulant use disorders: A meta-analysis of diffusion tensor imaging studies . Drug and Alcohol Dependence , 201 , 29 – 37 . 10.1016/j.drugalcdep.2019.03.023 31176066 PMC6660908

[b8] Beck , E. D. , & Jackson , J. J. ( 2022 ). A mega-analysis of personality prediction: Robustness and boundary conditions . Journal of Personality and Social Psychology , 122 ( 3 ), 523 – 553 . 10.1037/pspp0000386 35157487 PMC8867745

[b9] Bernoulli , D. ( 1954 ). Exposition of a new theory on the measurement of risk . Econometrica , 22 ( 1 ), 23 . 10.2307/1909829

[b10] Beshears , J. , Choi , J. J. , Laibson , D. , & Madrian , B. C. ( 2009 ). How are preferences revealed? Journal of Public Economics , 92 ( 8–9 ), 1787 – 1794 . 10.1016/j.jpubeco.2008.04.010 PMC3993927 24761048

[b11] Bringmann , L. F. , Elmer , T. , & Eronen , M. I. ( 2022 ). Back to basics: The importance of conceptual clarification in psychological science . Current Directions in Psychological Science , 31 ( 4 ), 340 – 346 . 10.1177/09637214221096485

[b12] Buckholtz , J. W. , Treadway , M. T. , Cowan , R. L. , Woodward , N. D. , Li , R. , Ansari , M. S. , Baldwin , R. M. , Schwartzman , A. N. , Shelby , E. S. , Smith , C. E. , Kessler , R. M. , & Zald , D. H. ( 2010 ). Dopaminergic network differences in human impulsivity . Science , 329 ( 5991 ), 532 – 532 . 10.1126/science.1185778 20671181 PMC3161413

[b13] Cao , Z. F. H. , Burdakov , D. , & Sarnyai , Z. ( 2011 ). Optogenetics: Potentials for addiction research . Addiction Biology , 16 ( 4 ), 519 – 531 . 10.1111/j.1369-1600.2011.00386.x 21929708 PMC5767107

[b14] Cartmell , S. C. , Tian , Q. , Thio , B. J. , Leuze , C. , Ye , L. , Williams , N. R. , Yang , G. , Ben-Dor , G. , Deisseroth , K. , Grill , W. M. , McNab , J. A. , & Halpern , C. H. ( 2019 ). Multimodal characterization of the human nucleus accumbens . NeuroImage , 198 , 137 – 149 . 10.1016/j.neuroimage.2019.05.019 31077843 PMC7341972

[b15] Casey , B. , Cannonier , T. , Conley , M. I. , Cohen , A. O. , Barch , D. M. , Heitzeg , M. M. , Soules , M. E. , Teslovich , T. , Dellarco , D. V. , Garavan , H. , Orr , C. A. , Wager , T. D. , Banich , M. T. , Speer , N. K. , Sutherland , M. T. , Riedel , M. C. , Dick , A. S. , Bjork , J. M. , Thomas , K. M. , … Dale , A. M. ( 2018 ). The Adolescent Brain Cognitive Development (ABCD) study: Imaging acquisition across 21 sites . Developmental Cognitive Neuroscience , 32 , 43 – 54 . 10.1016/j.dcn.2018.03.001 29567376 PMC5999559

[b16] Charness , G. , Gneezy , U. , & Imas , A. ( 2013 ). Experimental methods: Eliciting risk preferences . Journal of Economic Behavior and Organization , 87 , 43 – 51 . 10.1016/j.jebo.2012.12.023

[b17] Choe , A. S. , Stepniewska , I. , Colvin , D. C. , Ding , Z. , & Anderson , A. W. ( 2012 ). Validation of diffusion tensor MRI in the central nervous system using light microscopy: Quantitative comparison of fiber properties . NMR in Biomedicine , 25 ( 7 ), 900 – 908 . 10.1002/nbm.1810 22246940 PMC4818098

[b18] Chung , K. , & Deisseroth , K. ( 2013 ). CLARITY for mapping the nervous system . Nature Methods , 10 ( 6 ), 508 – 513 . 10.1038/nmeth.2481 23722210

[b19] Cohen , M. X. , Schoene-Bake , J. C. , Elger , C. E. , & Weber , B. ( 2009 ). Connectivity-based segregation of the human striatum predicts personality characteristics . Nature Neuroscience , 12 ( 1 ), 32 – 34 . 10.1038/nn.2228 19029888

[b20] Conrod , P. J. , O’Leary-Barrett , M. , Newton , N. , Topper , L. , Castellanos-Ryan , N. , Mackie , C. , & Girard , A. ( 2013 ). Effectiveness of a selective, personality-targeted prevention program for adolescent alcohol use and misuse . JAMA Psychiatry , 70 ( 334–342 ). 10.1001/jamapsychiatry.2013.651 23344135

[b21] Dalley , J. W. , & Robbins , T. W. ( 2017 ). Fractionating impulsivity: Neuropsychiatric implications . Nature Reviews Neuroscience , 18 ( 3 ), 158 – 171 . 10.1038/nrn.2017.8 28209979

[b22] Dutilh , G. , Vandekerckhove , J. , Ly , A. , Matzke , D. , Pedroni , A. , Frey , R. , Rieskamp , J. , & Wagenmakers , E. J. ( 2017 ). A test of the diffusion model explanation for the worst performance rule using preregistration and blinding . Attention, Perception, and Psychophysics , 79 ( 3 ), 713 – 725 . 10.3758/s13414-017-1304-y PMC5352774 28233280

[b23] Enkavi , A. Z. , Eisenberg , I. W. , Bissett , P. G. , Mazza , G. L. , Mackinnon , D. P. , & Marsch , L. A. ( 2019 ). Large-scale analysis of test— retest reliabilities of self-regulation measures . Proceedings of the National Academy of Sciences , 116 ( 12 ), 5472 – 5477 . 10.1073/pnas.1818430116 PMC6431228 30842284

[b24] Fields , R. D. ( 2015 ). A new mechanism of nervous system plasticity: Activity-dependent myelination . Nature Reviews Neuroscience , 16 ( 12 ), 756 – 767 . 10.1038/nrn4023 26585800 PMC6310485

[b25] Forkel , S. J. , Friedrich , P. , Thiebaut de Schotten , M. , & Howells , H. ( 2022 ). White matter variability, cognition, and disorders: A systematic review . Brain Structure and Function , 227 ( 2 ), 529 – 544 . 10.1007/s00429-021-02382-w 34731328 PMC8844174

[b26] Frey , R. , Pedroni , A. , Mata , R. , Rieskamp , J. , & Hertwig , R. ( 2017 ). Risk preference shares the psychometric structure of major psychological traits . Science Advances , 3 ( 10 ), 1 – 13 . 10.1126/sciadv.1701381 PMC5627985 28983511

[b27] Frey , R. , Richter , D. , Schupp , J. , Hertwig , R. , & Mata , R. ( 2021 ). Identifying robust correlates of risk preference: A systematic approach using specification curve analysis . Journal of Personality and Social Psychology , 120 ( 2 ), 538 – 557 . 10.1037/pspp0000287 32118465

[b28] Haber , S. N. , & Knutson , B. ( 2010 ). The reward circuit: Linking primate anatomy and human Imaging . Neuropsychopharmacology , 35 , 4 – 26 . 10.1038/npp.2009.129 19812543 PMC3055449

[b29] Hampton , W. H. , Alm , K. H. , Venkatraman , V. , Nugiel , T. , & Olson , I. R. ( 2017 ). Dissociable frontostriatal white matter connectivity underlies reward and motor impulsivity . NeuroImage , 150 , 336 – 343 . 10.1016/j.neuroimage.2017.02.021 28189592 PMC5494201

[b30] Hampton , W. H. , Hanik , I. M. , & Olson , I. R. ( 2019 ). Substance abuse and white matter: Findings, limitations, and future of diffusion tensor imaging research . Drug and Alcohol Dependence , 197 , 288 – 298 . 10.1016/j.drugalcdep.2019.02.005 30875650 PMC6440853

[b31] Janve , V. A. , Zu , Z. , Yao , S. Y. , Li , K. , Zhang , F. L. , Wilson , K. J. , Ou , X. , Does , M. D. , Subramaniam , S. , & Gochberg , D. F. ( 2013 ). The radial diffusivity and magnetization transfer pool size ratio are sensitive markers for demyelination in a rat model of type III multiple sclerosis (MS) lesions . NeuroImage , 74 , 298 – 305 . 10.1016/j.neuroimage.2013.02.034 23481461 PMC3995162

[b32] Jbabdi , S. , Lehman , J. F. , Haber , S. N. , & Behrens , T. E. ( 2013 ). Human and monkey ventral prefrontal fibers use the same organizational principles to reach their targets: Tracing versus tractography . Journal of Neuroscience , 33 ( 7 ), 3190 – 3201 . 10.1523/JNEUROSCI.2457-12.2013 23407972 PMC3602794

[b33] Joutsa , J. , Moussawi , K. , Siddiqi , S. H. , Abdolahi , A. , Drew , W. , Cohen , A. L. , Ross , T. J. , Deshpande , H. U. , Wang , H. Z. , Bruss , J. , Stein , E. A. , Volkow , N. D. , Grafman , J. H. , van Wijngaarden , E. , Boes , A. D. , & Fox , M. D. ( 2022 ). Brain lesions disrupting addiction map to a common human brain circuit . Nature Medicine , 28 ( 6 ), 1249 – 1255 . 10.1038/s41591-022-01834-y PMC9205767 35697842

[b34] Jung , W. H. , Lee , S. , Lerman , C. , & Kable , J. W. ( 2018 ). Amygdala functional and structural connectivity predicts individual risk tolerance . Neuron , 98 , 394 – 404.e4 . 10.1016/j.neuron.2018.03.019 29628186 PMC5910234

[b35] Kai , J. , Khan , A. , Haast , R. , & Lau , J. ( 2022 ). Mapping the subcortical connectome using in vivo diffusion MRI: Feasibility and reliability . NeuroImage , 262 , 15 . 10.1016/j.neuroimage.2022.119553 35961469

[b36] Karlsson Linnér , R. , Mallard , T. T. , Barr , P. B. , Sanchez-Roige , S. , Madole , J. W. , Driver , M. N. , Poore , H. E. , de Vlaming , R. , Grotzinger , A. D. , Tielbeek , J. J. , Johnson , E. C. , Liu , M. , Rosenthal , S. B. , Ideker , T. , Zhou , H. , Kember , R. L. , Pasman , J. A. , Verweij , K. J. H. , Liu , D. J. , … Dick , D. M. ( 2021 ). Multivariate analysis of 1.5 million people identifies genetic associations with traits related to self-regulation and addiction . Nature Neuroscience , 24 ( 10 ), 1367 – 1376 . 10.1038/s41593-021-00908-3 34446935 PMC8484054

[b37] Kotov , R. , Krueger , R. F. , & Watson , D. ( 2018 ). A paradigm shift in psychiatric classification: The hierarchical taxonomy of psychopathology (HiTOP) . World Psychiatry , 17 ( 1 ), 24 – 25 . 10.1002/wps.20478 29352543 PMC5775140

[b38] Krosnick , J. A. , Judd , C. M. , & Wittenbrink , B. ( 2005 ). The measurement of attitudes [Section: 2] . In D. Albarracin , B. Johnson , & M. Zanna (Eds.), The handbook of attitudes (pp. 21 – 76 ). Lawrence Erlbaum Associates Publishers . ISBN: 9781410612823 10.4324/9781410612823

[b39] Kruper , J. , Yeatman , J. D. , Richie-Halford , A. , Bloom , D. , Grotheer , M. , Caffarra , S. , Kiar , G. , Karipidis , I. I. , Roy , E. , & Rokem , A. ( 2021 ). Evaluating the reliability of human brain white matter tractometry . bioRxiv , 2021.02.24.432740. 10.1101/2021.02.24.432740 PMC8785971 35079748

[b40] Lazari , A. , & Lipp , I. ( 2021 ). Can MRI measure myelin? Systematic review, qualitative assessment, and meta-analysis of studies validating microstructural imaging with myelin histology . NeuroImage , 230 , 117744 . 10.1016/j.neuroimage.2021.117744 33524576 PMC8063174

[b41] Leong , J. K. , MacNiven , K. H. , Samanez-Larkin , G. R. , & Knutson , B. ( 2018 ). Distinct neural circuits support incentivized inhibition . NeuroImage , 178 , 435 – 444 . 10.1016/j.neuroimage.2018.05.055 29803959 PMC6398995

[b42] Leong , J. K. , Pestilli , F. , Wu , C. C. , Samanez-Larkin , G. R. , & Knutson , B. ( 2016 ). White-matter tract connecting anterior insula to nucleus accumbens correlates with reduced preference for positively skewed gambles . Neuron , 89 ( 1 ), 63 – 69 . 10.1016/j.neuron.2015.12.015 26748088 PMC4720154

[b43] Leuze , C. , Aswendt , M. , Ferenczi , E. , Liu , C. W. , Hsueh , B. , Goubran , M. , Tian , Q. , Steinberg , G. , Zeineh , M. M. , Deisseroth , K. , & McNab , J. A. ( 2017 ). The separate effects of lipids and proteins on brain MRI contrast revealed through tissue clearing . NeuroImage , 156 , 412 – 422 . 10.1016/j.neuroimage.2017.04.021 28411157 PMC5548623

[b44] Leuze , C. , Goubran , M. , Barakovic , M. , Aswendt , M. , Tian , Q. , Hsueh , B. , Crow , A. , Weber , E. M. , Steinberg , G. K. , Zeineh , M. , Plowey , E. D. , Daducci , A. , Innocenti , G. , Thiran , J. P. , Deisseroth , K. , & McNab , J. A. ( 2021 ). Comparison of diffusion MRI and CLARITY fiber orientation estimates in both gray and white matter regions of human and primate brain . NeuroImage , 228 , 117692 . 10.1016/j.neuroimage.2020.117692 33385546 PMC7953593

[b45] MacNiven , K. H. , Jensen , E. L. S. , Borg , N. , Padula , C. B. , Humphreys , K. , & Knutson , B. ( 2018 ). Association of neural responses to drug cues with subsequent relapse to stimulant use . JAMA Network Open , 1 ( 8 ), e186466 – e186466 . 10.1001/jamanetworkopen.2018.6466 30646331 PMC6324538

[b46] MacNiven , K. H. , Leong , J. K. , & Knutson , B. ( 2020 ). Medial forebrain bundle structure is linked to human impulsivity . Science Advances , 6 ( 38 ), 1 – 9 . 10.1126/sciadv.aba4788 PMC7494337 32938676

[b47] Mamerow , L. , Frey , R. , & Mata , R. ( 2016 ). Risk taking across the life span: A comparison of self-report and behavioral measures of risk taking . Psychology and Aging , 31 ( 7 ), 711 – 723 . 10.1037/pag0000124 27684105

[b48] Marek , S. , Tervo-Clemmens , B. , Calabro , F. J. , Montez , D. F. , Kay , B. P. , Hatoum , A. S. , Donohue , M. R. , Foran , W. , Miller , R. L. , Hendrickson , T. J. , Malone , S. M. , Kandala , S. , Feczko , E. , Miranda-Dominguez , O. , Graham , A. M. , Earl , E. A. , Perrone , A. J. , Cordova , M. , Doyle , O. , … Dosenbach , N. U. F. ( 2022 ). Reproducible brain-wide association studies require thousands of individuals . Nature , 603 ( 7902 ), 654 – 660 . 10.1038/s41586-022-04492-9 35296861 PMC8991999

[b49] Mata , R. , Frey , R. , Richter , D. , Schupp , J. , & Hertwig , R. ( 2018 ). Risk preference: A view from psychology . Journal of Economic Perspectives , 32 ( 2 ), 155 – 172 . 10.1257/jep.32.2.155 30203934

[b50] Mata , R. , Josef , A. K. , Samanez-Larkin , G. R. , & Hertwig , R. ( 2011 ). Age differences in risky choice: A meta-analysis . Annals of the New York Academy of Sciences , 1235 ( 1 ), 18 – 29 . 10.1111/j.1749-6632.2011.06200.x 22023565 PMC3332530

[b51] Mishra , S. ( 2014 ). Decision-making under risk: Integrating perspectives from biology, economics, and psychology . Personality and Social Psychology Review , 18 ( 3 ), 280 – 307 . 10.1177/1088868314530517 24769798

[b52] Moffitt , T. E. , Arseneault , L. , Belsky , D. , Dickson , N. , Hancox , R. J. , Harrington , H. , Houts , R. , Poulton , R. , Roberts , B. W. , Ross , S. , Sears , M. R. , Thomson , W. M. , & Caspi , A. ( 2011 ). A gradient of childhood self-control predicts health, wealth, and public safety . Proceedings of the National Academy of Sciences , 108 ( 7 ), 2693 – 2698 . 10.1073/pnas.1010076108 PMC3041102 21262822

[b53] Morales , A. M. , Jones , S. A. , Harman , G. , Patching-Bunch , J. , & Nagel , B. J. ( 2020 ). Associations between nucleus accumbens structural connectivity, brain function, and initiation of binge drinking . Addiction Biology , 25 ( 3 ), 1 – 9 . 10.1111/adb.12767 PMC7881761 31099090

[b54] Nichols , T. , & Holmes , A. ( 2002 ). Nonparametric permutation tests for functional neuroimaging: A primer with examples . Human Brain Mapping , 15 ( 1 ), 1 – 25 . 10.1002/hbm.1058 11747097 PMC6871862

[b55] Nigg , J. T. ( 2017 ). Annual Research Review: On the relations among self-regulation, self-control, executive functioning, effortful control, cognitive control, impulsivity, risk-taking, and inhibition for developmental psychopathology . Journal of Child Psychology and Psychiatry , 58 ( 4 ), 361 – 383 . 10.1111/jcpp.12675 28035675 PMC5367959

[b56] Ou , X. , Sun , S. W. , Liang , H. F. , Song , S. K. , & Gochberg , D. F. ( 2009 ). The MT pool size ratio and the DTI radial diffusivity may reflect the myelination in shiverer and control mice . NMR in Biomedicine , 22 ( 5 ), 480 – 487 . 10.1002/nbm.1358 19123230 PMC3711249

[b57] Pedroni , A. , Frey , R. , Bruhin , A. , Dutilh , G. , Hertwig , R. , & Rieskamp , J. ( 2017 ). The risk elicitation puzzle . Nature Human Behaviour , 1 ( 11 ), 803 – 809 . 10.1038/s41562-017-0219-x 31024120

[b58] Poldrack , R. A. , Monahan , J. , Imrey , P. B. , Reyna , V. , Raichle , M. E. , Faigman , D. , & Buckholtz , J. W. ( 2018 ). Predicting violent behavior: What can neuroscience add? Trends in Cognitive Sciences , 22 ( 2 ), 111 – 123 . 10.1016/j.tics.2017.11.003 29183655 PMC5794654

[b59] Rokem , A. , Yeatman , J. D. , Pestilli , F. , Kay , K. N. , Mezer , A. , van der Walt , S. , & Wandell , B. A. ( 2015 ). Evaluating the accuracy of diffusion MRI models in white matter . PLoS One , 10 ( 4 ), e0123272 . 10.1371/journal.pone.0123272 25879933 PMC4400066

[b60] Samanez-Larkin , G. R. , Levens , S. M. , Perry , L. M. , Dougherty , R. F. , & Knutson , B. ( 2012 ). Frontostriatal white matter integrity mediates adult age differences in probabilistic reward learning . Journal of Neuroscience , 32 ( 15 ), 5333 – 5337 . 10.1523/JNEUROSCI.5756-11.2012 22496578 PMC3744863

[b61] Schonberg , T. , Fox , C. R. , & Poldrack , R. A. ( 2011 ). Mind the gap: Bridging economic and naturalistic risk-taking with cognitive neuroscience . Trends in Cognitive Sciences , 15 ( 1 ), 11 – 19 . 10.1016/j.tics.2010.10.002 21130018 PMC3014440

[b62] Seaman , K. L. , Abiodun , S. , Fenn , Z. , Samanez-Larkin , G. R. , & Mata , R. ( 2022 ). Temporal discounting across adulthood: A systematic review and meta-analysis . Psychology and Aging , 37 ( 1 ), 111 – 124 . 10.1037/pag0000634 35113618 PMC8827494

[b63] Sherman , L. , Steinberg , L. , & Chein , J. ( 2018 ). Connecting brain responsivity and real-world risk taking: Strengths and limitations of current methodological approaches . Developmental Cognitive Neuroscience , 33 , 27 – 41 . 10.1016/j.dcn.2017.05.007 28774477 PMC5745301

[b64] Shivacharan , R. S. , Rolle , C. E. , Barbosa , D. A. N. , Cunningham , T. N. , Feng , A. , Johnson , N. D. , Safer , D. L. , Bohon , C. , Keller , C. , Buch , V. P. , Parker , J. J. , Azagury , D. E. , Tass , P. A. , Bhati , M. T. , Malenka , R. C. , Lock , J. D. , & Halpern , C. H. ( 2022 ). Pilot study of responsive nucleus accumbens deep brain stimulation for loss-of-control eating . Nature Medicine , 28 ( 9 ), 1791 – 1796 . 10.1038/s41591-022-01941-w PMC9499853 36038628

[b65] Simonsohn , U. , Simmons , J. P. , & Nelson , L. D. ( 2020 ). Specification curve analysis . Nature Human Behaviour , 4 ( 11 ), 1208 – 1214 . 10.1038/s41562-020-0912-z 32719546

[b66] Slovic , P. ( 1964 ). Assessment of risk taking behavior . Psychological Bulletin , 61 ( 3 ), 220 – 233 . 10.1037/h0043608 14130258

[b67] Song , S. K. , Sun , S. W. , Ramsbottom , M. J. , Chang , C. , Russell , J. , & Cross , A. H. ( 2002 ). Dysmyelination revealed through MRI as increased radial (but unchanged axial) diffusion of water . NeuroImage , 17 ( 3 ), 1429 – 1436 . 10.1006/nimg.2002.1267 12414282

[b68] Steegen , S. , Tuerlinckx , F. , Gelman , A. , & Vanpaemel , W. ( 2016 ). Increasing transparency through a multiverse analysis . Perspectives on Psychological Science , 11 ( 5 ), 702 – 712 . 10.1177/1745691616658637 27694465

[b69] Steinberg , L. ( 2013 ). The influence of neuroscience on US Supreme Court decisions about adolescents’ criminal culpability . Nature Reviews Neuroscience , 14 ( 7 ), 513 – 518 . 10.1038/nrn3509 23756633

[b70] Steiner , M. D. , Seitz , F. I. , & Frey , R. ( 2021 ). Through the window of my mind: Mapping information integration and the cognitive representations underlying self-reported risk preference . Decision , 8 ( 2 ), 97 – 122 . 10.1037/dec0000127

[b71] Stolp , H. B. , Ball , G. , So , P. W. , Tournier , J. D. , Jones , M. , Thornton , C. , & Edwards , A. D. ( 2018 ). Voxel-wise comparisons of cellular microstructure and diffusion-MRI in mouse hippocampus using 3D bridging of optically-clear histology with neuroimaging data (3D-BOND) . Scientific Reports , 8 ( 1 ), 1 – 12 . 10.1038/s41598-018-22295-9 29507311 PMC5838167

[b72] Suchting , R. , Beard , C. L. , Schmitz , J. M. , Soder , H. E. , Yoon , J. H. , Hasan , K. M. , Narayana , P. A. , & Lane , S. D. ( 2021 ). A meta-analysis of tract-based spatial statistics studies examining white matter integrity in cocaine use disorder . Addiction Biology , 26 ( 2 ), e12902 . 10.1111/adb.12902 32267062 PMC7541563

[b73] Tisdall , L. , Frey , R. , Horn , A. , Ostwald , D. , Horvath , L. , Pedroni , A. , Rieskamp , J. , Blankenburg , F. , Hertwig , R. , & Mata , R. ( 2020 ). Brain-outcome associations for risk taking depend on the measures used to capture individual differences . Frontiers in Behavioral Neuroscience , 14 , 587152 . 10.3389/fnbeh.2020.587152 33281576 PMC7705248

[b74] Tisdall , L. , MacNiven , K. , Padula , C. , Leong , J. , & Knutson , B. ( 2022 ). Brain tract structure predicts relapse to stimulant drug use . Proceedings of the National Academy of Sciences , 119 ( 26 ), e2116703119 . 10.1073/pnas.2116703119 PMC9245633 35727973

[b75] Tournier , J. D. , Calamante , F. , & Connelly , A. ( 2007 ). Robust determination of the fibre orientation distribution in diffusion MRI: Non-negativity constrained super-resolved spherical deconvolution . NeuroImage , 35 ( 4 ), 1459 – 1472 . 10.1016/j.neuroimage.2007.02.016 17379540

[b76] van den Bos , W. , Rodriguez , C. A. , Schweitzer , J. B. , & McClure , S. M. ( 2014 ). Connectivity strength of dissociable striatal tracts predict individual differences in temporal discounting . Journal of Neuroscience , 34 ( 31 ), 10298 – 10310 . 10.1523/JNEUROSCI.4105-13.2014 25080591 PMC4577570

[b77] Van Essen , D. C. , Smith , S. M. , Barch , D. M. , Behrens , T. E. , Yacoub , E. , & Ugurbil , K. ( 2013 ). The WU-Minn Human Connectome Project: An overview . NeuroImage , 80 , 62 – 79 . 10.1016/j.neuroimage.2013.05.041 23684880 PMC3724347

[b78] Vul , E. , & Pashler , H. ( 2012 ). Voodoo and circularity errors . NeuroImage , 62 ( 2 ), 945 – 948 . 10.1016/j.neuroimage.2012.01.027 22270348

[b79] Weiskopf , N. , Edwards , L. J. , Helms , G. , Mohammadi , S. , & Kirilina , E. ( 2021 ). Quantitative magnetic resonance imaging of brain anatomy and in vivo histology . Nature Reviews Physics , 3 , 570 – 588 . 10.1038/s42254-021-00326-1

[b80] Yarkoni , T. ( 2009 ). Big correlations in little studies. Inflated fMRI correlations reflect low statistical power - commentary on Vul et al. (2009) . Perspectives on Psychological Science , 4 ( 3 ), 294 – 298 . 10.1111/j.1745-6924.2009.01127.x 26158966

[b81] Yeatman , J. D. , Dougherty , R. F. , Ben-Shachar , M. , & Wandell , B. A. ( 2012 ). Development of white matter and reading skills . Proceedings of the National Academy of Sciences , 109 ( 44 ), E3045 – E3053 . 10.1073/pnas.1206792109 PMC3497768 23045658

[b82] Yeatman , J. D. , Dougherty , R. F. , Myall , N. J. , Wandell , B. A. , & Feldman , H. M. ( 2012 ). Tract profiles of white matter properties: Automating fiber-tract quantification . PLoS One , 7 ( 11 ), 1 – 15 . 10.1371/journal.pone.0049790 PMC3498174 23166771

