# Supplementary material for: Structural projections to the nucleus accumbens link to impulsive components of human risk preference
Source: Imaging Neurosci (Camb). 2024 Nov 5;2:imag-2-00344. doi: 10.1162/imag_a_00344 (PMC12290588; doi:10.1162/imag_a_00344)
Supplement: Supplementary Material [file imag_a_00344-supp.pdf]

SUPPLEMENTARY MATERIALS

Structural projections to the Nucleus Accumbens link to impulsive components of human risk preference

Loreen Tisdall, Kelly MacNiven, Josiah Leong, Renato Frey, Jörg Rieskamp, Ralph Hertwig, Brian Knutson, and Rui Mata

Supplementary Methods

Risk preference factors

As outcome variables we utilized risk preference factors that were independently derived in a previous study on the nature of risk preference (Frey et al., 2017). In that study, the authors collected a comprehensive battery of measures commonly used to capture individual differences in risk preference, including self-report measures to assess risk-taking propensity and frequency, as well as behavioral measures (Table S1). Note that some measures yielded multiple indices—e.g., different domains for propensity measures such as the socioeconomic panel or the domain-specific risk-attitude scale, and different performance indicators for decisions from experience paradigms—leading to a total number of 39 measures being entered into the model. To capture the psychometric structure of risk preference, Frey and colleagues (2017) adopted a bifactor model. Representative of reflective models, a bifactor model assumes that each observed indicator (e.g., risk preference measure) is influenced by two sources of variance, namely general as well as (domain) specific variance. Consequently, the contribution of both general and domain specific variance for each measure was estimated, resulting in a solution comprising one general and seven orthogonal domain-specific risk preference factors. In this study, we used the general and domain-specific risk preference factor values for BBRS participants who took part in the neuroimaging component of that study.

Table S1

Overview of measures adopted in the Basel-Berlin Risk Study.

Category	Measure	Reference
Propensity	Socioeconomic panel	(Falk et al., 2023)
Propensity	Domain-specific risk-attitude scale	(Weber et al., 2002)
Propensity	Gambling beliefs and attitudes survey	(Breen & Zuckerman, 1999)
Propensity	Personal risk inventory	(Hockey et al., 2000)
Propensity	Sensation seeking scale	(Zuckerman et al., 1964)
Propensity	Barratt impulsiveness scale	(Patton et al., 1995)
Frequency	Risky behaviors in the past month	(Weber et al., 2002)
Frequency	Alcohol use disorders identification test	(Allen et al., 2001)
Frequency	Fagerstroem test for nicotine dependence	(Heatherton et al., 1991)
Frequency	Pathological gambling	(Brodbeck et al., 2009)
Frequency	Drug abuse screening test	(Skinner, 1982)
Frequency	Cognitive appraisal of risky events	(Fromme et al., 1997)
Behavior	Balloon analogue risk task	(Lejuez et al., 2002)
Behavior	Decisions from experience	(Hertwig et al., 2004)
Behavior	Decisions from description	(Hertwig et al., 2004)
Behavior	Adaptive (economic) lotteries	(Rieskamp, 2008)
Behavior	Multiple price list	(Holt & Laury, 2002)
Behavior	Columbia card task	(Figner et al., 2009)
Behavior	Marbles task	(Dutilh & Rieskamp, 2016)
Behavior	Vienna risk-taking test traffic	(Hergovich et al., 2007)

DMRI metrics and outlier detection

In line with our main hypotheses and preregistration, we focused our analyses on fractional anisotropy (FA). FA is a commonly used DMRI metric indicating the extent to which water molecules are following a principal axonal direction, with values ranging between 0 (isotropic diffusion that is unrestricted or equally restricted in all directions) and 1 (anisotropic diffusion that is completely restricted and directed in one principal direction). As preregistered, we extended our focus to include analyses of a further DMRI metric, radial diffusivity (RD), given the latter's proposed direct link to axon myelination (Beard et al., 2019; Song et al., 2002). In contrast to FA, RD indicates water diffusivity perpendicular to the axon, such that lower values reflect a higher degree of directed diffusion. To ease interpretation and comparison of the different metrics, we inverted radial diffusivity as follows:

$$inverseRD = 1/RD$$

The resulting inverted RD metric (which we denote as 1/RD) can be interpreted akin to FA, with higher values representing more directed, restricted axonal diffusion, which in turn might indicate higher tract coherence.

To better understand the results obtained for the bilateral hypotheses (Table 1) with regard to potential laterality effects, we first explored inter-hemispheric correlations between DMRI metrics. In a second step, we performed additional analyses in which we examined associations separately for the left and right hemisphere of tracts for which we had bilateral hypotheses, including MPFC-NAcc, Amy-NAcc, and iVTA-NAcc.

Initial plotting of nodewise FA and 1/RD for all participants identified one subject with outlier DMRI metrics ($> \pm 3SD$) in an extended cluster of ~ 25 consecutive nodes in the left and right MPFC-NAcc tract. Further inspection confirmed sufficient overlap between this subject's MPFC VOI and the white matter mask, and also revealed no marked differences with regards to MPFC-NAcc tract location. We nevertheless excluded

this subject from all visualizations and analyses involving the MPFC-NAcc tract to avoid bias in our results stemming from this particular outlier. Analyses not involving the MPFC-NAcc tract were thus based on 125 subjects, and analyses involving MPFC-NAcc tract on 124 subjects (Figure S1).

Specification curve analysis

Specification curve analysis (SCA) (Simonsohn et al., 2020) has been used to probe the robustness of empirical findings in a variety of contexts (e.g., Frey et al., 2021; Lejarraga et al., 2019; Orben & Przybylski, 2019; Rohrer et al., 2017). In this study we used SCA to perform a principled, exhaustive quantification of the effect of tract coherence on different risk preference factors, while controlling for all possible combinations of covariates. The main motivation for this was that it allowed us to directly probe and visualize the convergence of tract effects as a function of the risk preference factor. We ended up running two SCAs, one for summary FA of the bilateral Amy-NAcc tract, and one for summary FA of the bilateral iVTA-NAcc tract. Specifically, each SCA included one predictor (summary tract metric), seven outcome variables (risk preference factors), and three covariates (age, gender, number of streamlines). The total number of specifications given the selected predictors, outcomes, and covariates was derived through additive combinations of predictor, outcomes, and covariates, with the restriction that a specification (i.e., a model) always contained the predictor, one outcome, and one unique combination of covariates, including no covariates. This approach resulted in $7 \times 2^3 = 56$ unique specifications. We estimated each specification via ordinary least-squares regression models using the R package *specr* (Masur & Scharkow, 2020) and entering scales variables for comparison of effect sizes. All specifications were based on data from 125 participants.

Exploratory node-wise associations

To better understand and qualify the results we obtained for the predicted associations between risk preference factors and brain tract structure, we explored the node-wise associations between the four brain tracts and all risk preference factors included

in the current analyses. In an effort to understand if unilateral associations may be driving the bilateral results, we analyzed associations separately for the left and right hemisphere of each tract, for both FA and 1/RD. We did not run any correction procedures, and instead report only the cluster extent and tract location for consecutive nodes significantly ($p < 0.05$) associated with a given risk preference factor.

Supplementary Results

Distributions of risk preference factor values

Shapiro Wilk tests indicated most of the risk preference factors to be normally distributed ($W > 0.98$, $p > 0.05$ for R, F1, F3, F4, and F6) in the study sample (Figure 1, panel A). Shapiro Wilk tests further indicated that the distribution of factors F2 and F5 deviated slightly from normality ($W = 0.97$, $p < 0.05$); the relatively large sample size may have contributed to minor deviations in the normality of F2 and F5 to result in significant test statistics. Visual inspection of density plots for F2 and F5 did not suggest substantial deviations from normality, thus we continued with parametric statistical analyses.

Tract coherence metrics

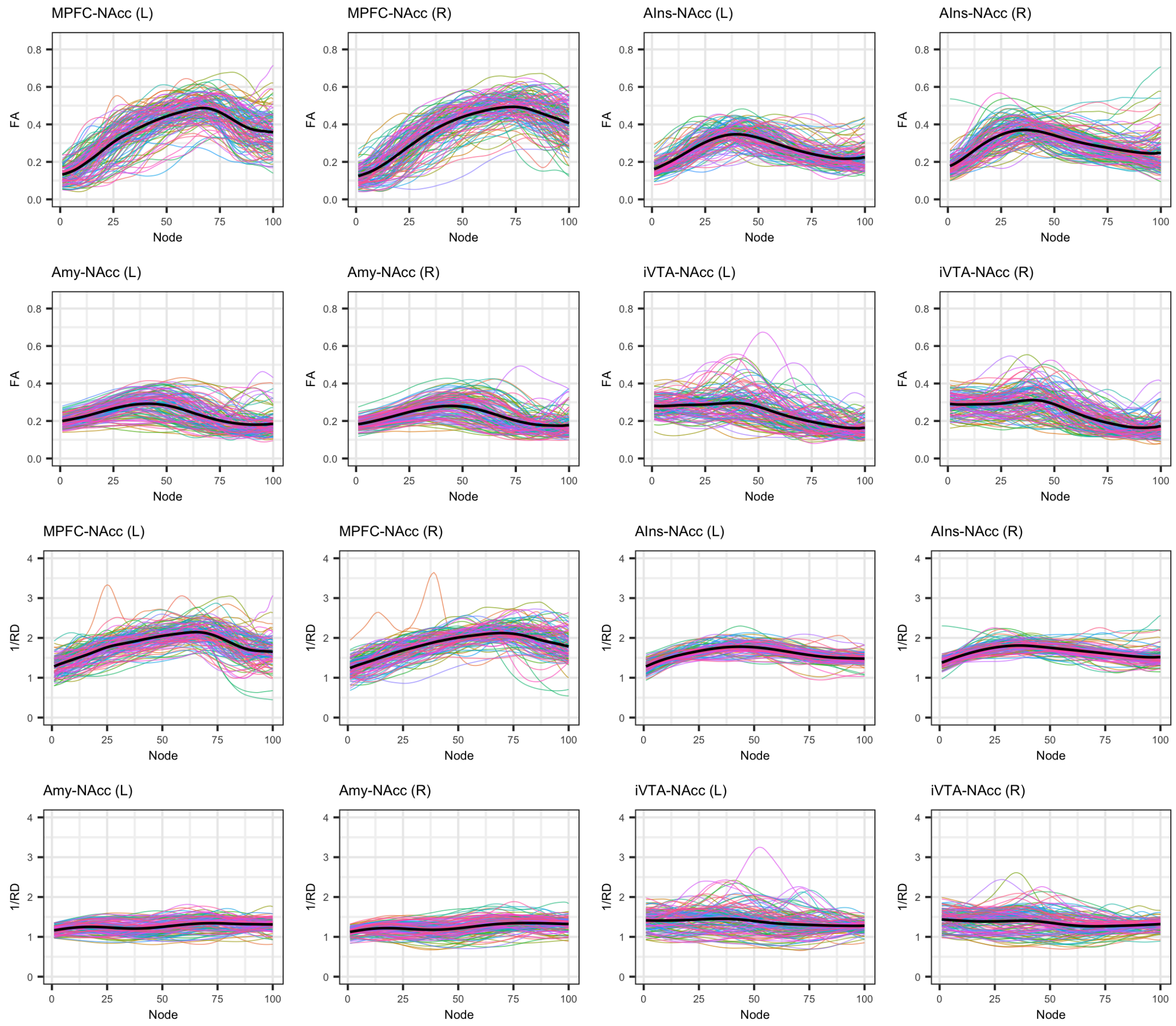

Figure S1

ConNAcctome tract profiles (after outlier removal). Plotted in color are subjects' individual tract profiles. Solid black lines represent mean sample coherence metrics across a given tract. The upper two rows show tract profiles for fractional anisotropy (FA), the lower two rows show tract profiles for inverse radial diffusivity (1/RD). On the x-axis, node 1 = MPFC/AIns/Amy/iVTA origin point, node 100 = NAcc endpoint.

Predicted associations between brain tract structure (1/RD) and risk preference factors

Table S2

Regression results for each hypothesis using 1/RD.

H	Factor	Tract (hemisphere)	# Observed/ # Required	Nodes	Predicted direction	Observed direction
1	R	MPFC-NAcc (lr)	5/27	32-36	−	+
2	R	AIns-NAcc (r)	12/26	82-93	−	N/A
3	R	Amy-NAcc (lr)	0/31	N/A	±	N/A
4	F4	Amy-NAcc (lr)	0/31	N/A	+	N/A
**5**	**F4**	**iVTA-NAcc (bl)**	**36/29**	**18-53**	−	−

Note. Note: H=Hypothesis; # Observed/# Required = Number of observed versus number of required consecutively significant (p=0.05) nodes; lr=bilateral; r=right; −=negative; +=positive; ±=bidirectional.

Specification curve analysis

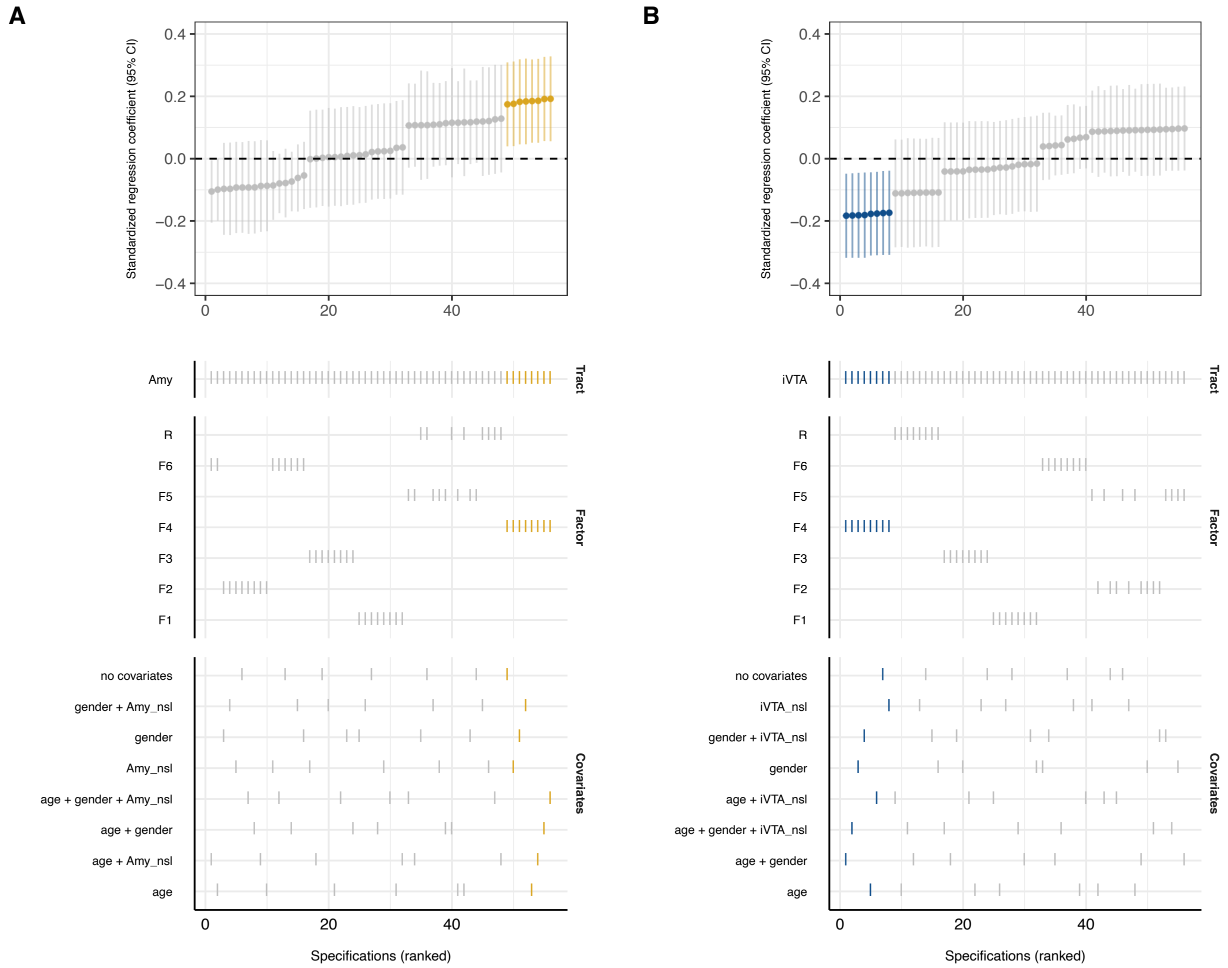

Figure S2

Specification curve analysis. ***A*** *Regression coefficients for bilateral Amy-NAcc tract FA.* ***B*** *Regression coefficients for bilateral iVTA-NAcc FA. Top panel displays the standardized regression coefficients (95% CI) for brain tract structure on latent risk preference factors, ordered by effect magnitude in ascending order. Tick marks in the lower panels indicate the exact specifications. Colors indicate significant ($p \leq 0.05$) positive (orange) or negative (blue) effects. nsl = number of streamlines.*

Effect of time interval on main results

Participants varied in the time between the laboratory session, where psychometric risk factors were derived, and the MRI session used to quantify structural projections to the NAcc. To rule out an effect of interval, we added interval (in months) into a supplementary set of SCAs (these additional analyses were not included in the preregistration). Note that models with interval as a covariate were based on 112 participants instead of 125 due to uncertainty in the exact session dates for 13 participants.

Overall, the results suggest that interval had no effect on the main results (Figure S3). The median effect sizes for Amy-NAcc FA (0.024) and iVTA-NAcc mean FA (-0.019) were in line with the median effect sizes reported for analyses without interval as an additional confound (0.024 and -0.022, respectively).

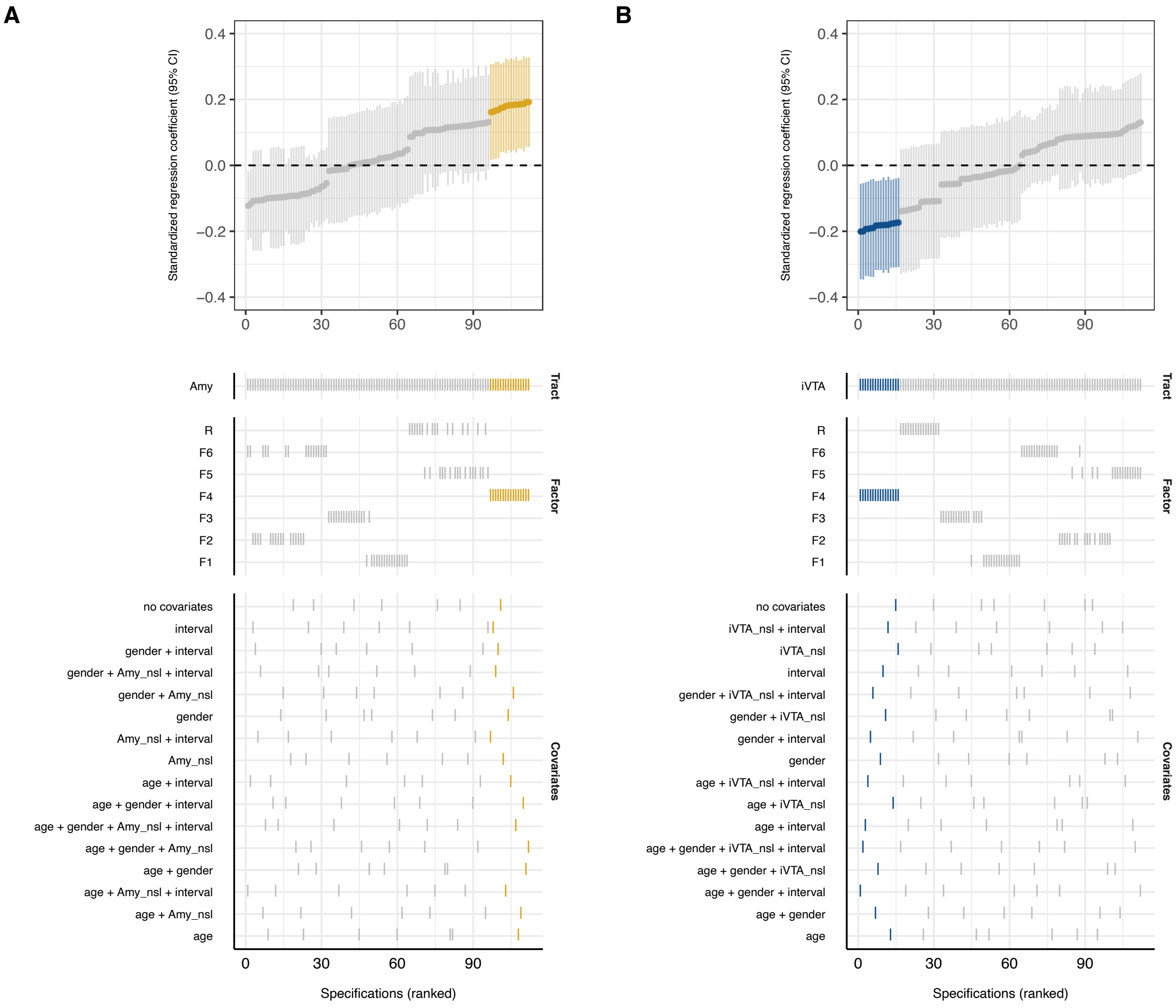

Figure S3

Specification curve analysis including interval as a covariate. ***A*** *Regression coefficients for bilateral Amy-NAcc tract FA.* ***B*** *Regression coefficients for bilateral iVTA-NAcc FA. Top panel displays the standardized regression coefficients (95% CI) for brain tract structure on latent risk preference factors, ordered by effect magnitude in ascending order. Tick marks in the lower panels indicate the exact specifications. Colors indicate significant ($p \leq 0.05$) positive (orange) or negative (blue) effects. nsl = number of streamlines.*

Exploratory node-wise associations

We explored the node-wise associations for all risk preference factors and tracts, for both hemispheres. As shown in Figure S4, these analyses suggested a strongly lateralized effect of MPFC-NAcc FA and 1/RD on the general risk preference factor R. Specifically, the association was pronounced for the left hemisphere but absent for the right hemisphere (Table S3). suggesting that the averaging across hemispheres resulted in a masking of the unilateral association. In contrast, the findings for associations between F4 and Amy-NAcc (FA) as well as F4 and iVTA-NAcc (FA, 1/RD) were evident in both hemispheres (Table S3).

Inspired by previous reports of the link between impulsivity and VTA-NAcc tract structure being specific to the inferior tract (MacNiven et al., 2020), we performed additional analyses for the superior VTA-NAcc tract. Our analyses replicated this finding, suggesting robust links between the impulsivity-capturing factor F4 and the inferior VTA-NAcc tract (iVTA), but not the superior VTA-NAcc tract (sVTA) (Table S3).

These exploratory analyses further suggested a strongly lateralized effect of MPFC-NAcc tract FA and 1/RD on F1, a factor capturing a range of concrete health-related behaviors, including alcohol consumption and nicotine dependence. Specifically, we found positive associations between nodes in an extended cluster along the right MPFC-NAcc tract and individual F1 scores (Figure S4, Table S3).

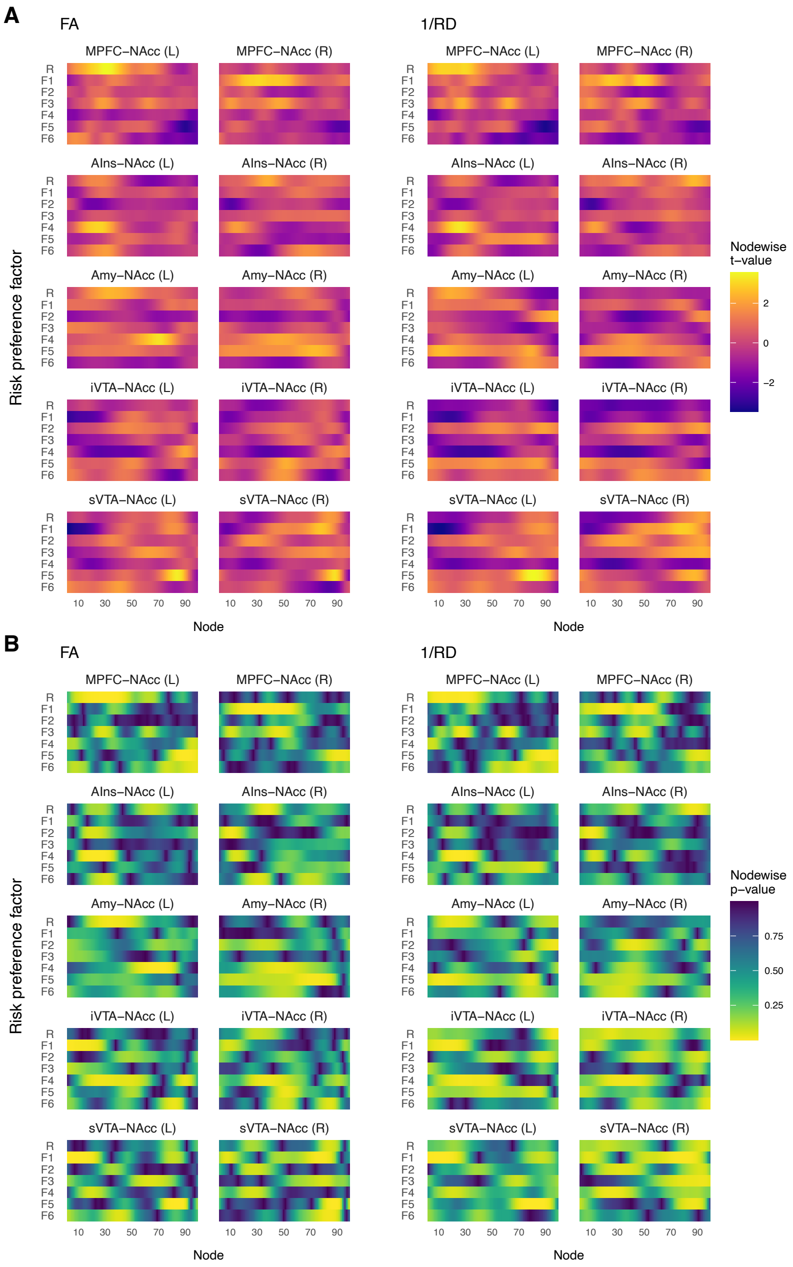

Figure S4

Heat maps of associations between risk preference factors and node-wise brain DMRI metrics. ***A*** *Node-wise t-values obtained from regression analyses (not controlling for confounding variables). Brighter yellow colors indicate larger positive t-values, darker purple colors indicate larger negative t-values.* ***B*** *Node-wise p-values obtained from regression analyses. Brighter yellow colors indicate smaller p-values.*

Table S3

Exploratory node-wise regression analyses (not controlling for confounds).

Factor	Tract (hemisphere)	Cluster extent FA (Nodes)	Cluster extent 1/RD (Nodes)
R	MPFC-NAcc (l)	36 (10-45)	35 (4-38)
R	MPFC-NAcc (r)	0	0
F1	MPFC-NAcc (l)	0	0
F1	MPFC-NAcc (r)	43 (15-57)	21 (16-36), 17 (41-57)
F4	Amy-NAcc (l)	26 (55-80)	0
F4	Amy-NAcc (r)	20 (38-57)	0
F4	iVTA-NAcc (l)	34 (19-52)	36 (16-51)
F4	iVTA-NAcc (r)	21 (28-48)	28 (22-49)
F4	sVTA-NAcc (l)	0	0
F4	sVTA-NAcc (r)	0	19 (17-35)

Note. Note: Cluster extent = Number of observed consecutive nodes associated with a given risk preference factor at $p = 0.05$; lr=bilateral; r=right; −=negative; +=positive; ±=bidirectional; iVTA = inferior VTA-NAcc tract; sVTA = superior VTA-NAcc tract.

Correlations between F4 and Barratt impulsiveness scale

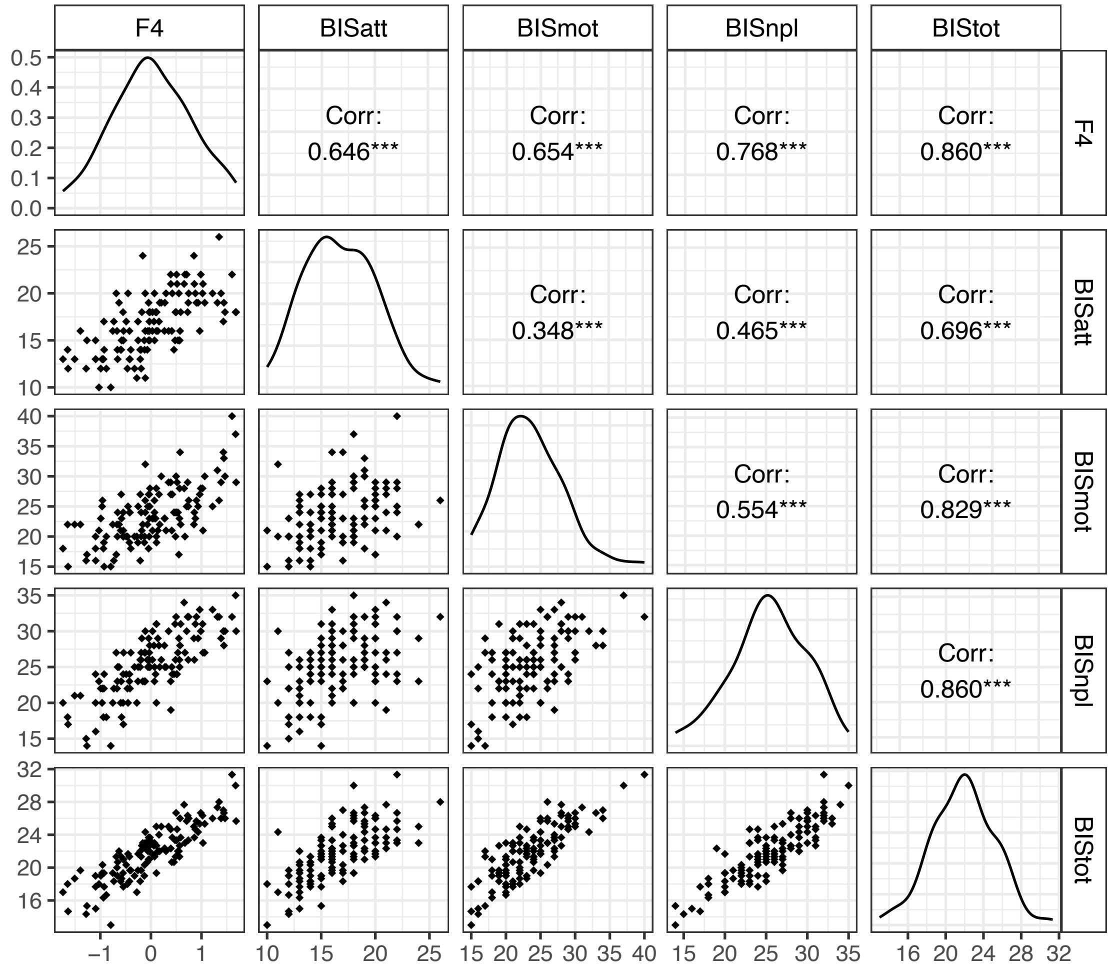

Figure S5

Scatterplot matrix of associations between impulsivity-factor F4 and Barratt impulsiveness scale. Correlation estimates are Pearson correlation coefficients. For the overall score (BIStot), we computed a simple average over the three subscales (BISatt = attention, BISmot = motor, BISnpl = non-planning).

References

Allen, J. P., Reinert, D. F., & Volk, R. J. (2001). The Alcohol use disorders identification test: An aid to recognition of alcohol problems in primary care patients. *Preventive Medicine*, *33*(5), 428–433. https://doi.org/10.1006/pmed.2001.0910

Beard, C. L., Schmitz, J. M., Soder, H. E., Suchting, R., Yoon, J. H., Hasan, K. M., Narayana, P. A., Moeller, F. G., & Lane, S. D. (2019). Regional differences in white matter integrity in stimulant use disorders: A meta-analysis of diffusion tensor imaging studies. *Drug and Alcohol Dependence*, *201*(February), 29–37. https://doi.org/10.1016/j.drugalcdep.2019.03.023

Breen, R. B., & Zuckerman, M. (1999). 'Chasing' in gambling behavior: Personality and cognitive determinants. *Personality and Individual Differences*, *27*(6), 1097–1111. https://doi.org/10.1016/S0191-8869(99)00052-5

Brodbeck, J., Znoj, H., & Dürrenberger, S. (2009). Prevalence rates of at risk, problematic and pathological gambling in Switzerland. *European Journal of Psychiatry*, *23*(2), 67–75. https://doi.org/10.7892/BORIS.34175

Dutilh, G., & Rieskamp, J. (2016). Comparing perceptual and preferential decision making. *Psychonomic Bulletin & Review*, *23*(3), 723–737. https://doi.org/10.3758/s13423-015-0941-1

Falk, A., Becker, A., Dohmen, T., Huffman, D., & Sunde, U. (2023). The preference survey module: A validated instrument for measuring risk, time, and social preferences. *Management Science*, *69*(4), 1935–1950.

Figner, B., Mackinlay, R. J., Wilkening, F., & Weber, E. U. (2009). Affective and deliberative processes in risky choice: Age differences in risk taking in the Columbia Card Task. *Journal of Experimental Psychology: Learning, Memory, and Cognition*, *35*(3), 709–730.

Frey, R., Pedroni, A., Mata, R., Rieskamp, J., & Hertwig, R. (2017). Risk preference shares the psychometric structure of major psychological traits. *Science Advances*, *3*(10), 1–13. https://doi.org/10.1126/sciadv.1701381

Frey, R., Richter, D., Schupp, J., Hertwig, R., & Mata, R. (2021). Identifying robust correlates of risk preference: A systematic approach using specification curve analysis. *Journal of Personality and Social Psychology*, *120*(2), 538–557. https://doi.org/10.1037/pspp0000287

Fromme, K., Katz, E. C., & Rivet, K. (1997). Outcome expectancies and risk-taking behavior. *Cognitive Therapy and Research*, *21*(4), 421–442.

Heatherton, T. F., Kozlowski, L. T., Frecker, R. C., & Fagerstrom, K.-O. (1991). The Fagerström Test for Nicotine Dependence: A revision of the Fagerstrom Tolerance Questionnaire. *British Journal of Addiction*, *86*(9), 1119–1127. https://doi.org/10.1111/j.1360-0443.1991.tb01879.x

Hergovich, A., Arendasy, M. E., Sommer, M., & Bognar, B. (2007). The Vienna Risk-Taking Test - Traffic: A new measure of road traffic risk-taking. *Journal of Individual Differences*, *28*(4), 198–204. https://doi.org/10.1027/1614-0001.28.4.198

Hertwig, R., Barron, G., Weber, E. U., & Erev, I. (2004). Decisions from experience and the effect of rare events in risky choice. *Psychological Science*, *15*(8), 534–539. http://www.jstor.org/stable/40064012

Hockey, G. R. J., John Maule, A., Clough, P. J., & Bdzola, L. (2000). Effects of negative mood states on risk in everyday decision making. *Cognition and Emotion*, *14*(6), 823–855. https://doi.org/10.1080/02699930050156654

Holt, C. A., & Laury, S. K. (2002). Risk aversion and incentive effects. *American Economic Review*, *92*(5), 1644–1655.

Lejarraga, T., Frey, R., Schnitzlein, D. D., & Hertwig, R. (2019). No effect of birth order on adult risk taking. *Proceedings of the National Academy of Sciences*, *116*(13), 6019–6024. https://doi.org/10.1073/pnas.1814153116

Lejuez, C. W., Read, J. P., Kahler, C. W., Richards, J. B., Ramsey, S. E., Stuart, G. L., Strong, D. R., & Brown, R. A. (2002). Evaluation of a behavioral measure of risk taking: The Balloon Analogue Risk Task (BART). *Journal of Experimental Psychology. Applied*, *8*(2), 75–84. https://doi.org/10.1037/1076-898X.8.2.75

MacNiven, K. H., Leong, J. K., & Knutson, B. (2020). Medial forebrain bundle structure is linked to human impulsivity. *Science Advances*, *6*(38), 1–9. https://doi.org/10.1126/sciadv.aba4788

Masur, P. K., & Scharkow, M. (2020). Specr: Conducting and visualizing specification curve analyses. https://cran.r-project.org/web/packages/specr/index.html

Orben, A., & Przybylski, A. K. (2019). The association between adolescent well-being and digital technology use. *Nature Human Behaviour*, *3*(2), 173–182. https://doi.org/10.1038/s41562-018-0506-1

Patton, J. H., Stanford, M. S., & Barratt, E. S. (1995). Factor structure of the Barratt Impulsiveness Scale. *Journal of Clinical Psychology*, *51*(6), 768–774. http://homepages.se.edu/cvonbergen/files/2013/01/Factor-Structure-of-the-Barratt-Impulsiveness-Scale.pdf

Rieskamp, J. (2008). The probabilistic nature of preferential choice. *Journal of Experimental Psychology: Learning, Memory, and Cognition*, *34*(6), 1446–1465. https://doi.org/10.1037/a0013646

Rohrer, J. M., Egloff, B., & Schmukle, S. C. (2017). Probing birth-order effects on narrow traits using specification-curve analysis. *Psychological Science*, *28*(12), 1821–1832. https://doi.org/10.1177/0956797617723726

Simonsohn, U., Simmons, J. P., & Nelson, L. D. (2020). Specification curve analysis. *Nature Human Behaviour*, *4*(11), 1208–1214. https://doi.org/10.1038/s41562-020-0912-z

Skinner, H. A. (1982). The drug abuse screening test. *Addictive Behaviors*, *7*(4), 363–371. https://doi.org/10.1016/0306-4603(82)90005-3

Song, S. K., Sun, S. W., Ramsbottom, M. J., Chang, C., Russell, J., & Cross, A. H. (2002). Dysmyelination revealed through MRI as increased radial (but unchanged axial) diffusion of water. *NeuroImage*, *17*(3), 1429–1436. https://doi.org/10.1006/nimg.2002.1267

Weber, E. U., Blais, A.-R., & Betz, N. E. (2002). A domain-specific risk-attitude scale: Measuring risk perceptions and risk behaviors [ISBN: 1099-0771]. *Journal of Behavioral Decision Making*, *15*(August), 263–290. https://doi.org/10.1002/bdm.414

Zuckerman, M., Kolin, E. A., Price, L., & Zoob, A. (1964). Development of a sensation-seeking scale. *Journal of Consulting Psychology*, *28*(6), 477–482.
